# A randomized trial of AmBisome monotherapy and AmBisome and miltefosine combination to treat visceral leishmaniasis in HIV co-infected patients in Ethiopia

**DOI:** 10.1371/journal.pntd.0006988

**Published:** 2019-01-17

**Authors:** Ermias Diro, Severine Blesson, Tansy Edwards, Koert Ritmeijer, Helina Fikre, Henok Admassu, Aderajew Kibret, Sally J. Ellis, Clelia Bardonneau, Eduard E. Zijlstra, Peninah Soipei, Brian Mutinda, Raymond Omollo, Robert Kimutai, Gabriel Omwalo, Monique Wasunna, Fentahun Tadesse, Fabiana Alves, Nathalie Strub-Wourgaft, Asrat Hailu, Neal Alexander, Jorge Alvar

**Affiliations:** 1 Leishmaniasis Research and Treatment Centre, University of Gondar, Gondar, Ethiopia; 2 Research & Development Department, Drugs for Neglected Diseases *initiative*, Geneva, Switzerland; 3 MRC Tropical Epidemiology Group, London School of Hygiene and Tropical Medicine, London, United Kingdom; 4 Médecins sans Frontières, Amsterdam, the Netherlands; 5 Abdurafi Health Centre, Médecins sans Frontières, Abdurafi, Ethiopia; 6 Drugs for Neglected Diseases *initiative*, Nairobi, Kenya; 7 Neglected Tropical Diseases, Federal Ministry of Health, Addis Ababa, Ethiopia; 8 Department of Microbiology, Immunology, and Parasitology, Addis Ababa University, Addis Ababa, Ethiopia; Hospital Universitário Professor Edgard Santos, BRAZIL

## Abstract

**Background:**

Visceral leishmaniasis (VL) in human immunodeficiency virus (HIV) co-infected patients requires special case management. AmBisome monotherapy at 40 mg/kg is recommended by the World Health Organization. The objective of the study was to assess if a combination of a lower dose of AmBisome with miltefosine would show acceptable efficacy at the end of treatment.

**Methodology/Principal findings:**

An open-label, non-comparative randomized trial of AmBisome (30 mg/kg) with miltefosine (100 mg/day for 28 days), and AmBisome monotherapy (40 mg/kg) was conducted in Ethiopian VL patients co-infected with HIV (NCT02011958). A sequential design was used with a triangular continuation region. The primary outcome was parasite clearance at day 29, after the first round of treatment. Patients with clinical improvement but without parasite clearance at day 29 received a second round of the allocated treatment. Efficacy was evaluated again at day 58, after completion of treatment.

Recruitment was stopped after inclusion of 19 and 39 patients in monotherapy and combination arms respectively, as per pre-specified stopping rules. At D29, intention-to-treat efficacy in the AmBisome arm was 70% (95% CI 45–87%) in the unadjusted analysis, and 50% (95% CI 27–73%) in the adjusted analysis, while in the combination arm, it was 81% (95% CI 67–90%) and 67% (95% CI 48–82%) respectively. At D58, the adjusted efficacy was 55% (95% CI 32–78%) in the monotherapy arm, and 88% (95% CI 79–98%) in the combination arm.

No major safety concerns related to the study medication were identified. Ten SAEs were observed within the treatment period, and 4 deaths unrelated to the study medication.

**Conclusions/Significance:**

The extended treatment strategy with the combination regimen showed the highest documented efficacy in HIV-VL patients; these results support a recommendation of this regimen as first-line treatment strategy for HIV-VL patients in eastern Africa.

**Trial registration number:**

www.clinicaltrials.gov NCT02011958.

## Author summary

Visceral Leishmaniasis is a complex parasitological disease and is particularly challenging to treat in patients coinfected with human immunodeficiency virus (HIV). Antimonial drugs used in first-line treatments for immunocompetent patients in eastern Africa are more toxic in immunocompromised patients. In 2010, a WHO expert committee recommended a lipid formulation of amphotericin B as first line treatment for HIV/VL co-infected patients, based on a single clinical trial conducted in Spain and empirical information obtained from scattered case reports using AmBisome (liposomal amphotericin B). In addition, Médecins Sans Frontières began a compassionate use regimen combining AmBisome and miltefosine a in a treatment centre in Northwest Ethiopia with encouraging results. Here, we report the results of a trial to assess the efficacy and safety of both the currently internationally recommended treatment of AmBisome monotherapy and the new AmBisome-miltefosine combination regimen, in Ethiopian patients. The results of this trial show that one course of treatment with either regimen could be insufficient to clear parasites in a high proportion of patients and that an extended treatment strategy, of administrating a second course of treatment, could lead to a high parasite clearance rate in patients treated with the combination regimen.

## Introduction

Human immunodeficiency virus (HIV) affects visceral leishmaniasis (VL) by increasing its incidence, altering its clinical manifestation and severity, and, more importantly, by worsening treatment outcomes and relapse rates [1,2]. VL is the second most deadly protozoan infection after malaria. HIV-VL co-infection has been observed in at least 35 countries on four continents [3–6]. Following the introduction of highly active anti-retroviral therapy (HAART), the incidence of VL in HIV patients has decreased in most settings [7]. Northwest Ethiopia has the highest burden globally, with HIV rates among VL patients ranging between 20–40% [2,3]. Typically, young male seasonal workers migrate to the lowlands to harvest crops, sleep in improvised shelters, and are exposed to the bites of sand flies. In addition, migrant workers are at high risk of HIV infection [2,8,9].

The current WHO recommended regimen is infusion of amphotericin B lipid formulations 3–5 mg/kg daily or intermittently up to a total dose of 40 mg/kg [10], despite the absence of proper evaluation in most endemic areas. The Ethiopian National Guidelines (2013) [11] recommend liposomal amphotericin B and sodium stibogluconate (SSG) as the first and second line treatments for HIV-VL patients. While effectiveness studies at 40 mg/kg AmBisome are lacking, effectiveness at 30 mg/kg was less than 60% among HIV co-infected patients [12]. SSG has poor effectiveness (43%–70%) and considerable toxicity, with an increased risk of death in HIV co-infected patients [13,14]. Thus, there is an urgent need for a better treatment approach to VL in HIV co-infected patients.

Preventing recurrence of disease is another important factor, as relapse cases are even more difficult to cure, consequently becoming reservoirs of the parasite and playing a role in transmission [15]. Negative aspirate after treatment is the best predictor of absent/delayed relapse. Surviving HIV patients develop some tolerance to the parasite. The annual VL relapse rate among HAART-taking HIV patients in Northwest Ethiopia was more than 60% if their CD4 count was less than 100 cells/μl [16]. A recent cohort study in Northwest Ethiopia demonstrated 71% disease-free survival one year after VL treatment using pentamidine as secondary prophylaxis [17].

This clinical trial was conducted primarily to evaluate the day 29 efficacy of a combination of AmBisome (30 mg/kg) with miltefosine (100 mg/day for 28 days) and AmBisome monotherapy (40 mg/kg) for VL in HIV co-infected patients in Northwest Ethiopia. Secondary objectives were to assess efficacy at day 58 and the safety of the regimens. A long-term evaluation of relapse-free survival up to one-year follow-up (Day 390), including an estimate of the relapse rate in those receiving pentamidine as a prophylactic treatment, will be reported in another publication.

## Methods

### Ethics statement

This study was conducted according to principles of the Helsinki declaration, Good Clinical Practice (GCP) rules, and local regulations. The Protocol and the Informed Consent Sheet were revised and approved by the University of Gondar Institutional Review Board, the Ethiopian National Research Ethics Review Committee, the Médecins Sans Frontières Ethics Review Board, the London School of Hygiene and Tropical Medicine Research Ethics Committee, the Antwerp University Hospital Ethics Committee, and the Prince Leopold Institute of Tropical Medicine Institutional Review Board. Approval from the Food, Medicine and Healthcare Administration and the Control Authority of Ethiopia was also obtained before inclusion of the patients. Patients were included after completion of a written informed consent process.

### Study design

**Fig 1:**
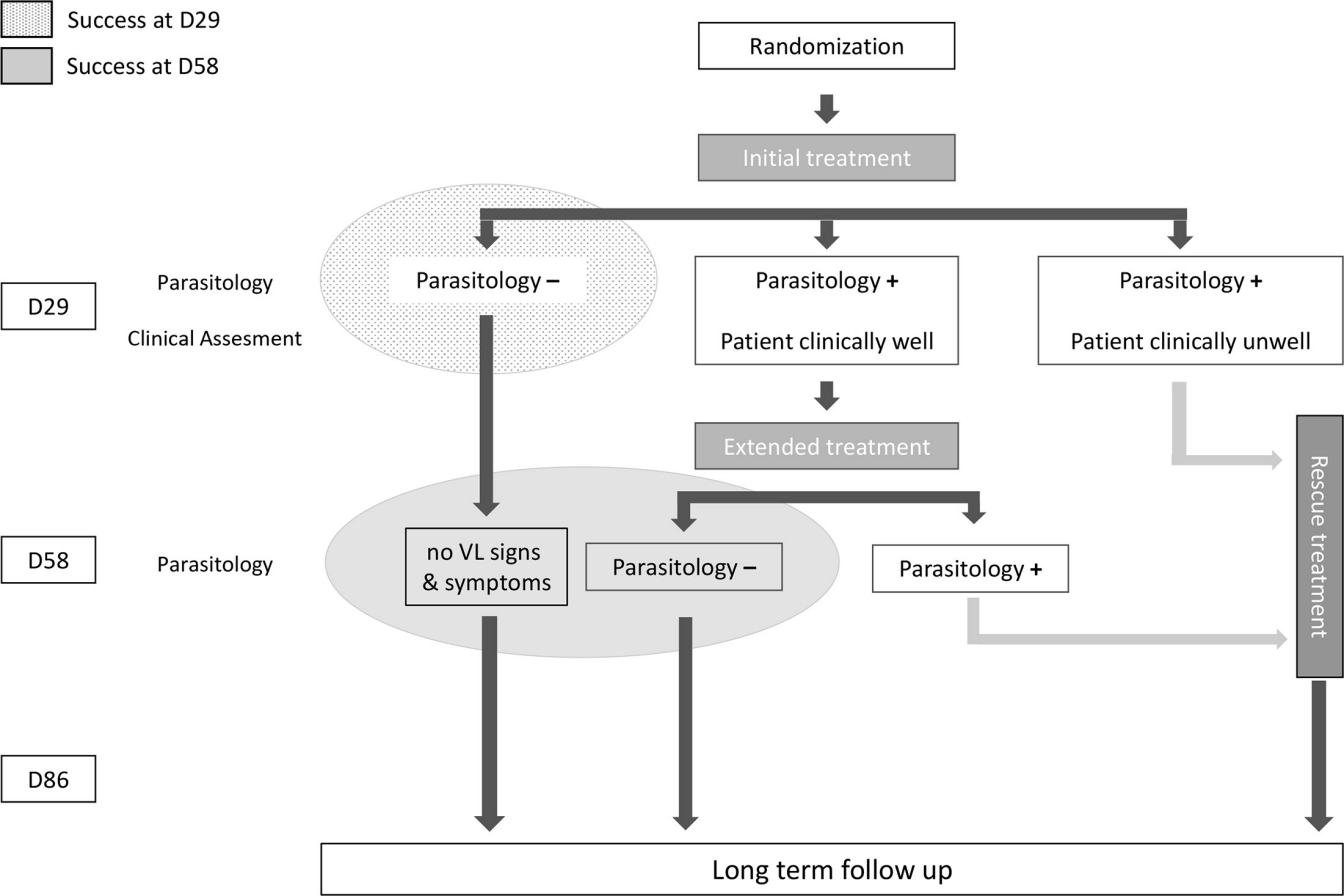
Treatment strategy.

**Fig 2:**
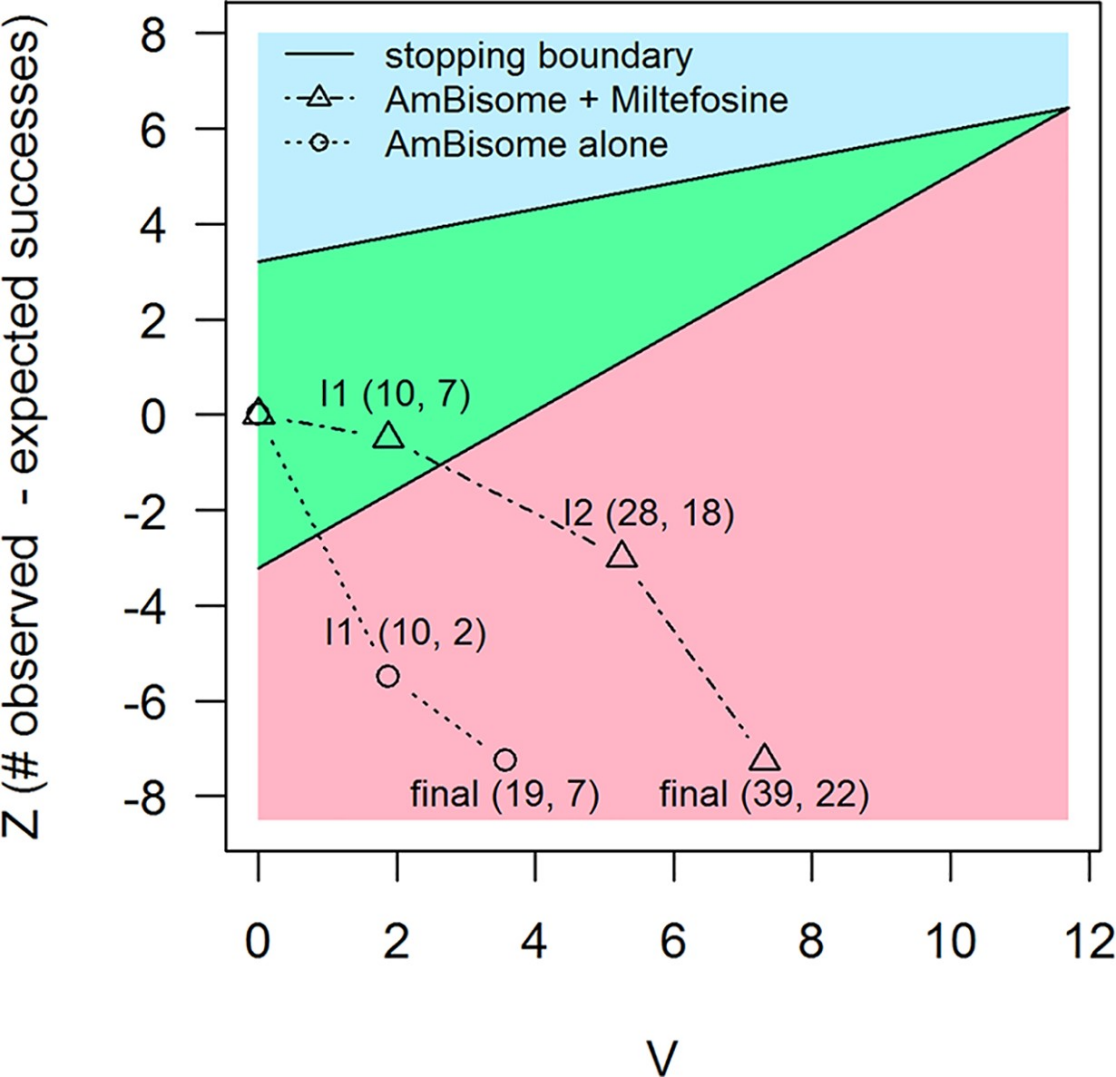
Sequential analysis. The vertical axis, (*Z*), is the number of observed treatment successes minus the number of expected successes. The higher the value represented on that vertical axis, the more favorable (better efficacy observed). The horizontal axis *(V)* is proportional to the number of patients who, over time, have been evaluated at day 29. Hence, over time, each arm’s data points extend to the right. Each arm starts inside the triangle (green area) and recruitment is stopped on crossing either the upper (blue area) or the lower boundary (pink area). Recruitment to the AmBisome monotherapy arm was stopped after the first interim analysis (I1) and to the combination arm after the second interim analysis (I2). Also shown are the final results for each arm, including those who had not reached the primary endpoint (day29) when the interim analysis was performed and the recruitment was stopped. These final results take the trajectory of each arm well away from the triangular boundary, which is known as ‘over-run'. The two numbers after each label are the numbers of patients included in that analysis and the number of these with treatment success at day 29.

This study, registered on www.clinicaltrials.gov (NCT02011958), included two components. The first component was a non-comparative randomized open-label clinical trial to evaluate the end of treatment efficacy and safety of two treatment regimens for VL in HIV co-infected patients (Fig 1). This component used a sequential design, in which the sample size is not fixed in advance but depends on the accruing endpoint data. More specifically, we used a triangular continuation region which is a graphical representation of the stopping rules of the trial [18,19] (Fig 2). The results cannot easily be interpreted without reference to the graphical representation. The trial design allows for regular interim analyses, and hence possible stopping, after every 10 patients, based on acceptable or unacceptable efficacy values set in the protocol. This design allows recruitment to be stopped as patients are being evaluated if the observed efficacy is too low (lack of promise), i.e. crossing the lower boundary of the triangular region (pink area), or sufficiently high (promise), i.e. crossing the upper boundary (blue area). Otherwise recruitment continues, corresponding to the interior of the triangular continuation region in the graphical representation (green area). In more technical terms, the null hypothesis was that the proportion of patients reaching negative parasitology at day 29 (*p*) is less than or equal to a value *p*_0,_ which we set to 75%. A test statistic falling outside the continuation region in the graphical representation would lead to a decision to stop recruitment. If the upper boundary is crossed during an interim analysis, then the null hypothesis is rejected and we conclude that *p*>75%. Crossing the lower boundary implies that the null hypothesis (proportion reaching negative parasitology ≤75%) is not rejected and there is a specified power to exclude a proportion reaching negative parasitology of *p*_*a*_, for which we chose a value of 90%. Less formally, on crossing the upper boundary, the trial should be stopped for promise (efficacy above 75%), and on crossing the lower boundary it should be stopped for lack of promise (efficacy below 90%). The type I error rate and power of the study were pre-specified as 5% and 95%, respectively (*α* = *β* = 0·05). Interim analyses were specified after every 10 patients in each arm had reached the Day 29 endpoint. An independent Data Safety Monitoring Board (DSMB) evaluated the results and confirmed decisions to continue or stop with recruitment in each arm. Based on the sequential trial design and the parameters described above, the maximum sample size per arm was 66. The second component included a follow-up for 12 months with secondary prophylaxis for severely immunocompromised patients. Results will be described in a separate publication.

Patients were recruited at two facilities in Ethiopia, the Leishmaniasis Research and Treatment Centre, located within the teaching hospital of the University of Gondar, and the Abdurafi Health Centre, located in a rural setting on the border with Sudan and supported by Médecins Sans Frontières. These two sites are the main VL treatment centers in Ethiopia. On average 400 and 800 VL patients are managed every year respectively at these two centers.

### Participants

Patients were diagnosed for VL and for HIV as per the Ethiopian national guidelines [11]. Upon presentation of clinical signs and symptoms of VL (e.g. irregular fever for more than 2 weeks, hepatomegaly and/or splenomegaly, weight loss), the patients were diagnosed by identification of the *Leishmania* parasite by microscopy in tissue aspirate (spleen aspirate was the preferred methodology, or bone marrow aspirate in case of contra-indication e.g. bleeding tendency, platelets below 40,000/mm^3^, haemoglobin below 5 g/dL). Patients were eligible regardless of whether this was the first episode of VL (primary case) or whether it was a relapse case with single or multiple relapses. The parasite strains were not identified, but according to epidemiological reports [20,21], the circulating parasite causing visceral leishmaniasis in Ethiopia is *Leishmania donovani*. HIV status is routinely determined by two rapid tests followed by a third confirmatory test in case of discrepancy. Within the trial, it was reconfirmed using an enzyme immunoassay (ImmunoComb II HIV 1&2 BiSpot, Orgenics Ltd.).

### Randomization and masking

Subjects were allocated to treatment using random block sizes, stratified by site (Gondar & Abdurafi) and by patient type (whether the VL episode at screening was a primary or relapse case).

The randomization list was prepared by the data management team. Site investigators were blinded to block sizes. Randomization codes were prepared in sealed, sequentially numbered, opaque envelopes and were under the control of the site investigator.

Patients and treating physicians were not masked to study treatment due to the considerable differences in the administration of the treatment arms (different dosing schedule of an infused treatment plus oral administration).

### Procedures

Patients received in-patient treatment. Liposomal amphotericin B (AmBisome, Gilead Inc.) was stored and reconstituted as per the manufacturer’s recommendations. In the monotherapy arm, a dose of 40 mg/kg total dose of AmBisome (adapted from WHO recommendations) was administered by intra-venous (IV) slow infusion of 5 mg/kg on days 1 to 5, 10, 17, and 24.

In the combination arm, a 30 mg/kg total dose of AmBisome was administered according to the following schedule: IV slow infusion of 5 mg/kg on days 1, 3, 5, 7, 9, and 11; with a miltefosine (Paladin Therapeutics, Inc.) 50 mg capsule orally twice a day for 28 days (all patients included were above 25 kg).

For both arms, a pre-testing dose of 1 mg of AmBisome was administered to patients before treatment to rule out allergic reactions, as recommended in the AmBisome Summary of Product Characteristics.

An initial assessment of cure was conducted at D29, through clinical and parasitological examination (spleen or bone marrow aspiration). Patients with negative parasitology were considered cured of VL and started the follow-up period (Fig 1). Patients who had clinically improved but still had detectable parasites at D29 were given extended treatment using the same regimen to which they had been randomized. Patients with detectable parasites at D29, and who were clinically unwell, and patients with parasites at D58 were given a rescue medication of the clinician’s choice (Fig 1).

Once the patients had a negative parasitology result, they started a follow-up period of one year (up to D390) to assess long-term relapse-free survival and safety (not reported here).

### Outcomes

The primary endpoint was parasitological clearance at day 29 (D29), which was defined as absence of parasites in tissue aspirate at D29 (bone marrow or spleen, with spleen aspirate as the preferred option). Treatment failure at D29 was defined as presence of parasites at the D29 assessment, or death prior to the D29 assessment, or no clinical response to treatment requiring rescue medication on or before D29.

A secondary endpoint to assess treatment outcome after extended treatment was defined as efficacy at day 58 (D58). D58 treatment success was defined as: (i) being parasite free at D29 and no recurrence of symptoms by D58 or (ii) being parasite free at D58 after extended treatment. Thus, D58 failures were patients who (i) received rescue treatment prior to, or at, the D58 visit, or (ii) were confirmed to be parasite positive at D58 or (iii) died up to D58. A patient with detectable parasites at D29 who then received extended treatment would be a treatment failure at D29 but a success at D58 if no parasites were detected at D58.

All adverse events (AEs) and serious adverse events (SAEs) were captured up to one month after the last dose of study medication (D58 if patients received one round of treatment or D86 if patients received a second round). Grading of the severity of the events was based on Common Terminology Criteria for Adverse Events (CTCAE), Version 4.0 [22].

Safety laboratory assessments were performed at baseline, D3, D10, D29, and D58, and during follow-up as needed. CD4 count was measured at baseline, on D29, and within one month of reaching negative parasitology. HIV viral load was measured at baseline and every 6 months as per routine practice, samples were sent to central regional or national reference laboratories. Results of the viral load were often not available in time to support HIV case management.

### Statistical analysis

Data capture and management utilized OpenClinica and Stata software [23]. Data analysis was performed using Stata, version 14.

The intention to treat (ITT) population was pre-specified as *primary analysis population* for the sequential interim analyses of treatment success at D29. Both ITT and per-protocol (PP) populations for treatment success at D29 and D58 were used for final analyses of treatment success at D29 and D58. ITT was considered *primary analysis population*. Interim analyses took place each time 10 patients per arm had reached the D29 primary endpoint assessment. Decision making was based on the position of the test statistic relative to the triangular continuation region, as described above [18]. Recruitment and randomization of new patients were not stopped during interim analysis, but were stopped on the recommendation of the DSMB when the interim analysis showed that the boundary of the continuation region had been crossed.

In a sequential trial, recruitment is stopped when a pre-specified difference is reached in the accruing data. In the current trial, this difference is between the observed and expected numbers of treatment successes. Stopping when a difference of interest has been achieved implies a risk that the final results are a “random high” [24]. In other words, the results will tend to be more extreme than if the sample size had been fixed in advance. To allow for this, specific analysis methods are required. In particular, simple maximum likelihood estimates—which in the current trial would be simple proportions—in general suffer from ‘substantial bias’ [25], in sequential trials. Here, the pre-specified approach, taking into account the sequential design, was to use point and interval estimates of efficacy obtained at D29, as per Whitehead [26]. Because this approach assumes that the final data lie close to the boundary of the continuation region (Fig 2), an additional post-hoc analysis, as per Liu *et al*. [27], was done of D29 efficacy to allow for this and for ‘over-run’, i.e. inclusion, in the final analysis, of people who had been randomized to the trial before the final interim analysis, but whose D29 data were not then available for inclusion.

The sequential stopping rule applied only as far as the primary outcome at D29. Hence, the D58 outcome is subject to a mixture of sequential and classical follow-up. To take this into account, the probability of treatment success at D58 was estimated by a probability tree method previously developed for this purpose [28].

All adverse events were coded according to Medical Dictionary for Regulatory Activities (MedDRA, Version 14.0). Safety outcomes were the number (%) of patients experiencing a serious adverse event (SAE) at any time during the trial or follow-up, the number (%) of patients experiencing an adverse event (AE) between Day 1 of initial treatment and up to one month after completing treatment, and the incidence of adverse drug reactions (ADRs) by preferred MedDRA term estimated as the number (%) of patients experiencing at least one ADR for each MedDRA lower level preferred term. We also report the rate of ADR accounting for time at risk based on the number of rounds of treatment.

CD4 counts were compared between time points by paired *t* test.

## Results

**Fig 3:**
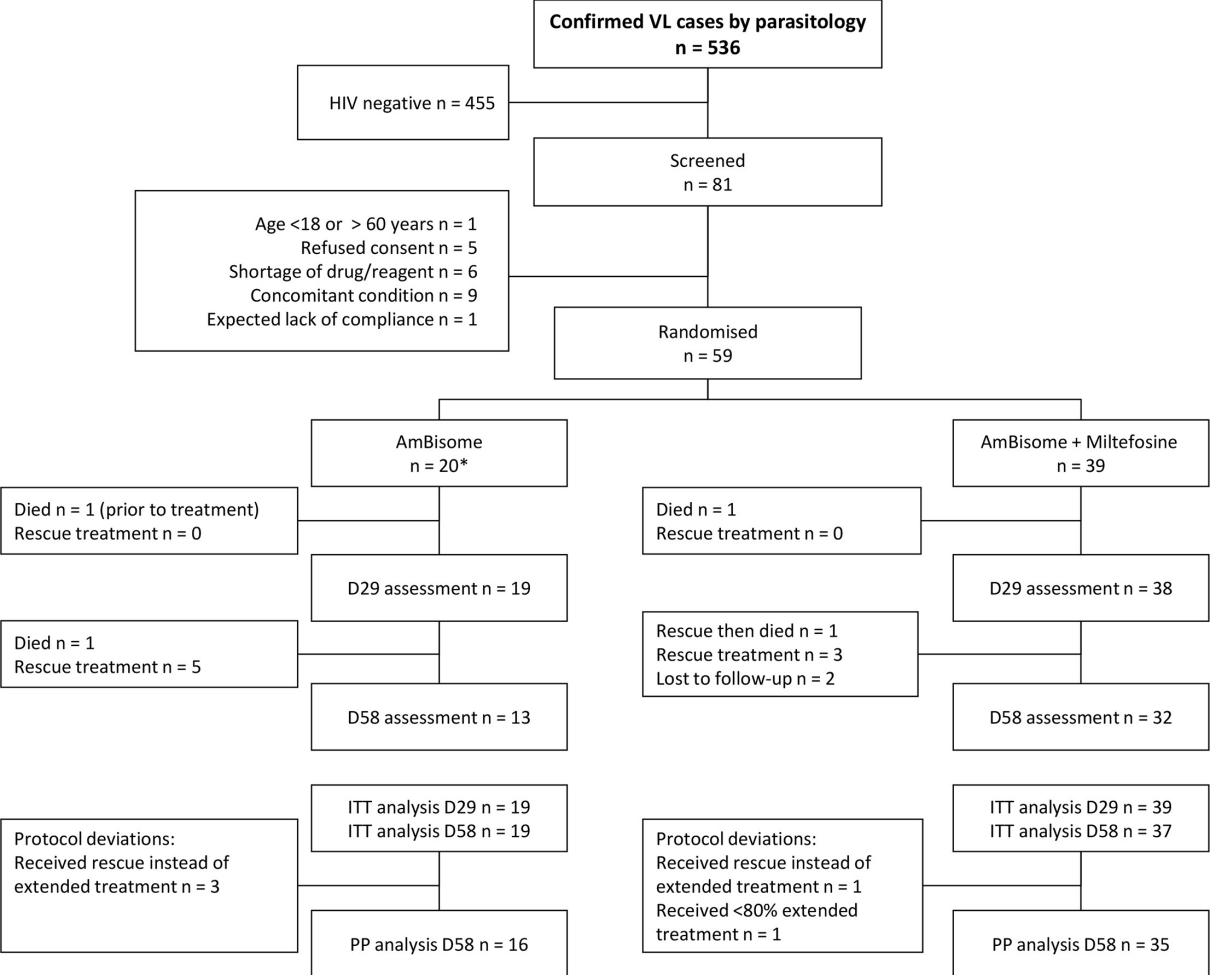
Trial participant flow.

Patients were recruited between 14 August 2014 and 18 August 2015. Out of the 536 parasitologically confirmed VL patients (Fig 3), 81 were HIV positive according to the Ethiopian national HIV testing algorithm and confirmatory test and 59 patients were enrolled.

Both arms had a similar distribution of baseline characteristics, including body mass index, VL status, CD4 count, and prior antiretroviral treatment (Tables 1 and 2).

**Table 1:** Baseline characteristics.

		AmBisome	AmBisome + miltefosine
Number of patients		19	39
Age (years)	mean (SD)	37 (8)	33 (5)
	median (range)	38 (27–51)	33 (21–45)
Sex, n (%)	Male / Female	19 (100) / 0 (0)	38 (97) / 1 (3)
Patient Type, n (%)	Primary / Relapse	8 (44) / 10 (56)	18 (50) / 18 (50)
Site, n (%)	Gondar / Abdurafi	10 (53) / 9 (47)	20 (51) / 19 (49)
Spleen size^a^ (cm):	mean (SD)	8·1 (5)	7·3 (3)
	median (range)	6 (4–22)	7 (3–15)
Body Mass Index status, n (%)	Severely underweight (BMI<15)	0 (0)	5 (13)
	Underweight (15·0≤BMI≤18·4)	12 (63)	25 (64)
	Normal (18·5≤BMI≤24·9)	7 (37)	8 (21)
	Overweight (BMI≥25)	0 (0)	1 (3)
Type of Tissue aspirate	Spleen aspirate	18	36
	Bone marrow aspirate	1	3
Parasite count^b^	+4 to +6, n (%)	16 (84)	29 (75)
	+1 to +3, n (%)	2 (11)	7 (18)
Haemoglobin (g/dL)	mean (SD)	8·8 (2)	8·1 (1·9)
	median (range)	9·1 (4·9–12)	8·1 (3·6–12·3)
WBC (x10^3^/μL)	mean (SD)	2·2 (0·9)	2 (1·1)
	median (range)	2·2 (0·8–4·6)	1·7 (0·4–4·3)
Platelets (x10^3^/μL)	mean (SD)	112 (54)	126 (66)
	median (range)	95 (50–217)	117 (39–315)
SGOT /AST (U/L)	mean (SD)	51 (33)	58 (72)
	median (range)	42 (15–119)	38 (21–472)
SGPT /ALT (U/L)	mean (SD)	39 (38)	35 (50)
	median (range)	24 (3–157)	23 (7–325)
Creatinine (mg/dL)	mean (SD)	0·9 (0·2)	1 (0·2)
	median (range)	0·9 (0·7–1·3)	1 (0·6–1·4)
Potassium (mmol/L)	mean (SD)	3·7 (1)	3·4 (0·8)
	median (range)	3·8 (2·3–5·4)	3·5 (2·1–4·9)
CD4 (cells/μL)	median (range)	69 (30–121)	54 (33–96)

^a^ Below the costal margin

^b^ Parasite count is not reported for those tested by bone marrow aspiration

**Table 2:** HIV parameters and antiretroviral (ART) regimen at baseline.

		AmBisome		AmBisome + miltefosine	
		ART at baseline		ART at baseline	
		No	Yes	No	Yes
Nb of patients		6	13	11	28
CD4 count (cells/μl)	<50	2 (33)	6 (46)	6 (55)	11 (39)
	50–99	4 (67)	2 (15)	3 (27)	11 (39)
	100 to 199	0 (0)	5 (38)	2 (18)	5 (18)
	200–349	0 (0)	0 (0)	0 (0)	1 (4)
	≥350	0 (0)	0 (0)	0 (0)	0 (0)
	Median (IQR)	70 (30–77)	50 (44–129)	45 (33–97)	56 (31–96)
Viral Load (copies per ml)	<150 –undetectable	0 (0)	7 (54)	0 (0)	9 (32)
	150 to <3 log_10_	0 (0)	3 (23)	0 (0)	4 (14)
	≥3 to <4 log_10_	0 (0)	0 (0)	0 (0)	2 (7)
	≥4 to <5 log_10_	0 (0)	1 (8)	1 (9)	4 (14)
	≥5 to <6 log_10_	3 (50)	2 (15)	4 (36)	4 (14)
	≥6 log_10_	3 (50)	0 (0)	5 (45)	3 (11)
	missing	0 (0)	0 (0)	1 (9)	2 (7)
	Median (IQR)^1^	6.51 log_10_ (5·68–6·92 log_10_)	150 (150–926)	5·98 log_10_ (5·30–6·38 log_10_)	869 (150–5·26 log_10_)

^1^ Values less than detection threshold, or less than 150 copies/ml have been set to 150 copies/ml.

Prior to D29 there were no protocol deviations, thus the ITT populations were identical to the PP. At D58, the PP population excluded 5 patients with major protocol deviations (Fig 3). There were no missing outcome data. One patient died after randomization before receiving any treatment and was excluded from all analyses.

In interim analyses, both arms crossed the lower side of the boundary (Fig 2), indicating treatment success of less than 90% at D29. The AmBisome monotherapy crossed first, based on data from 10 patients, and the combination arm crossed the boundary based on data from 20 patients.

In the AmBisome arm, there were only 7 treatment successes out of 19 subjects in the final data. Stopping in this way, for a difference of a given magnitude, is characteristic of a sequential trial and required particular statistical methods, as explained above. This pre-specified analysis gives an estimated efficacy of 70% (95% CI 45–87%) at D29 in the AmBisome arm. However, this does not take account of the substantial over-run to the lower side of the boundary, visible in Fig 2 (see the Fig 2 legend for an explanation of over-run). After accounting for this, the estimated efficacy was lower, at 50% (27–73%). In the combination arm, the estimated success rate is 81% from the pre-specified efficacy analysis (67–90%), and with an adjusted efficacy of 67% (48–82%), taking account of over-run (Table 3).

**Table 3:** Efficacy (as per sequential analysis).

	AmBisome			AmBisome + miltefosine		
	N	Treatment success, n	%, 95% CI*	N	Treatment success, n	%, 95% CI*
**Cumulative analysis (D29)**						
ITT & PP [26]	19	7	70 (45–87)	39	22	81 (67–90)
ITT &PP (over run) [27]	19	7	50 (27–73)	39	22	67 (48–82)
**Cumulative analysis (D58)**						
ITT [26]	19	9	68 (44–91)	37	31	93 (87–99)
PP [26]	16	9	70 (46–94)	35	31	95 (90–100)
ITT (over run) [27]	19	9	55 (32–78)	37	31	88 (79–98)
PP (over run) [27]	16	9	59 (35–83)	35	31	91 (82–100)

* The pre-specified analysis approach accounts for features of the trial design and therefore the appropriate point estimate of efficacy is not simply a ratio of the number of treatment successes to the number randomized.

Efficacy was also estimated at Day 58 (Table 3) to evaluate the success of the extended treatment strategy. Using the over-run adjusted D29 analysis as inputs, the D58 ITT efficacy was 55% (95% CI 32–78%) in the monotherapy arm, and the PP efficacy was 59% (35–83%). The same analysis in the combination arm gave 88% (79–98%) and 91% (81–100%) for ITT and PP respectively. These D58 efficacy estimates are higher than those at D29 because improvement in status between these two time points was more likely than deterioration, thanks to prolongation of the treatment. If the over-run phenomenon is not considered, D58 efficacy estimates are slightly higher in both arms, as was the case for D29 (Table 3).

All patients experienced at least one AE (Table 4). A higher percentage of patients experienced at least one ADR in the combination arm than in the monotherapy arm. However, the rate of ADR was similar between arms, after accounting for time-at-risk for those receiving one or two rounds of treatment (Table 5). AEs and ADRs were mostly mild or moderate. ADRs occurring in more than 10% of patients were dyspepsia, gastritis, vomiting mainly related to miltefosine, increased blood creatinine, and hypokalaemia related to AmBisome. Two hypokalaemia events (one per arm) were severe in intensity as per CTCAE [22] criteria (between 2·5 and 3 mmol/L) and required supplementation. All ADRs were considered expected as per the reference documentation for each drug [29,30]. Ten SAEs were reported during the treatment phase (Table 6), including four deaths, mostly due to infectious events (sepsis, strongyloidiasis hyperinfection syndrome, meningitis/encephalitis due either to toxoplasma or tuberculosis). One patient died from pancreatitis/renal failure related to rescue treatment with sodium stibogluconate and paromomycin, along with ARV drugs, a combination known to be toxic in HIV-VL co-infected patients [14].

**Table 4:** Safety during the treatment phase.

	AmBisome	AmBisome + miltefosine
	N = 19	N = 39
**Number (%) of patients with at least one SAE**		
Total*	2 (10)	8 (21)
Related to study drugs (ADR)	0 (0)	0 (0)
Unrelated to study drugs	2 (10)	8 (21)
**Number (%) of patients with at least one AE (whether serious or not)**		
Total*	19 (100)	39 (100)
Related to study drugs (ADR)	12 (63)	33 (85)
Unrelated to study drugs	18 (95)	33 (85)
**Number (%) of patients with at least one ADR (whether serious or not) by intensity**				
1 Mild	11 (58)	22 (56)
2 Moderate	3 (16)	15 (38)
3 Severe	1 (5)	1 (3)
4 Life-threatening	0 (0)	0 (0)
5 Death	0 (0)	0 (0)
**Number of ADRs per patient**		
Median	1	1
Range	0–5	0–5
Total person days at risk**	1262	2534
Unadjusted rate per day (95% CI)	0.018 (0.011–0.026)	0.026 (0.019–0.032)

AE = adverse event, SAE = serious adverse event, ADR = adverse drug reaction; Events reported here occurred during the treatment phase i.e. from day 1 to day 58 for patients with one round of treatment, day 1 to day 86 for those on extended treatment

* these rows do not necessarily add to the total number of patients because a single patient may have events in multiple rows

** Time at risk is from day 1 to day 86 for those on extended treatment, otherwise from day 1 to day 58. For those who died before these limits, their time at risk was from day 1 until their death.

**Table 5:** Incidence of ADRs due to AmBisome or miltefosine. Incidence>10% indicated in bold.

Number of patients with ADR, n (%)	AmBisome N = 19	AmBisome + miltefosine N = 39				Total N = 58
		Possibly related to:				
		AmBisome	Miltefosine	Either	Total	
Gastrointestinal disorders						
Abdominal pain	0 (0)	0 (0)	1 (3)	0 (0)	1 (3)	1 (2)
Diarrhoea	1 (5)	0 (0)	0 (0)	0 (0)	0 (0)	1 (2)
**Dyspepsia**	**2 (11)**	0 (0)	**6 (15)**	2 (5)	**8 (21)**	**10 (17)**
**Gastritis**	0 (0)	0 (0)	**9 (23)**	0 (0)	**9 (23)**	**9 (16)**
Glossitis	1 (5)	0 (0)	0 (0)	0 (0)	0 (0)	1 (2)
Nausea	0 (0)	0 (0)	0 (0)	1 (3)	1 (3)	1 (2)
Peptic ulcer	1 (5)	0 (0)	2 (5)	0 (0)	2 (5)	3 (5)
Stomatitis	1 (5)	0 (0)	0 (0)	0 (0)	0 (0)	1 (2)
Vomiting	**3 (16)**	0 (0)	**9 (23)**	2 (5)	**11 (28)**	**14 (24)**
General disorders and administration site conditions						
Pain	1 (5)	0 (0)	0 (0)	0 (0)	0 (0)	1 (2)
Infections and infestations						
Folliculitis	0 (0)	0 (0)	0 (0)	1 (3)	1 (3)	1 (2)
Investigations						
**Blood creatinine increased**	**5 (26)**	**10 (26)**	0 (0)	1 (3)	**11 (28)**	**16 (28)**
Metabolism and nutrition disorders						
**Hypokalaemia**	**4 (21)**	**6 (15)**	0 (0)	0 (0)	**6 (15)**	**10 (17)**
Musculoskeletal and connective tissue disorders						
Back pain	1 (5)	1 (3)	1 (3)	0 (0)	1 (3)	2 (3)
Neck pain	1 (5)	0 (0)	0 (0)	0 (0)	0 (0)	1 (2)
Polyarthritis	0 (0)	0 (0)	0 (0)	1 (3)	1 (3)	1 (2)
Nervous system disorders						
Cluster headache	0 (0)	0 (0)	0 (0)	1 (3)	1 (3)	1 (2)
Headache	0 (0)	1 (3)	0 (0)	0 (0)	1 (3)	1 (2)
Skin and subcutaneous tissue disorders						
Pruritus	0 (0)	0 (0)	0 (0)	1 (3)	1 (3)	1 (2)
Rash papular	0 (0)	0 (0)	0 (0)	1 (3)	1 (3)	1 (2)

**Table 6:** Serious adverse events.

ID	MedDRA preferred term	Grade^a^	Relation to study drug^b^	Day of onset	Outcome
**AmBisome monotherapy**					
303	Sepsis	3	1	3	Resolved
307	Malnutrition	3	1	30	Death
	Decubitus ulcer	3	1	38	
	Pneumonia	4	1	39	
	Sepsis	5	1	39	
**AmBisome + miltefosine**					
112	Anaemia	4	1	48	Resolved
113	Strongyloidiasis	5	1	61*	Death
115	Anaemia	4	1	10	Resolved
207	Anaemia	3	1	3	Resolved
408	Post herpetic neuralgia	2	1	10	Resolved
410	Toxicity to various agents^c^	5	1	33	Death
411	Encephalitis	5	1	15	Death
	Meningitis	5	1	15	
415	Pulmonary tuberculosis	3	1	20	Unknown

^a^Mild (grade 1), moderate (grade 2), severe (grade 3), life threatening (grade 4), death (grade 5)

^b^1 = Not related 2 = Possibly related

^c^ Toxicity was related to sodium stibogluconate and paromomycin administered as rescue treatment and to ART drugs (patient received sequentially zidovudine/lamivudine/nevirapine and tenofovir/lamivudine/nevirapine)

* This death occurred slightly after the treatment phase recording period but is reported here as first symptoms were reported during the period.

**Fig 4:**
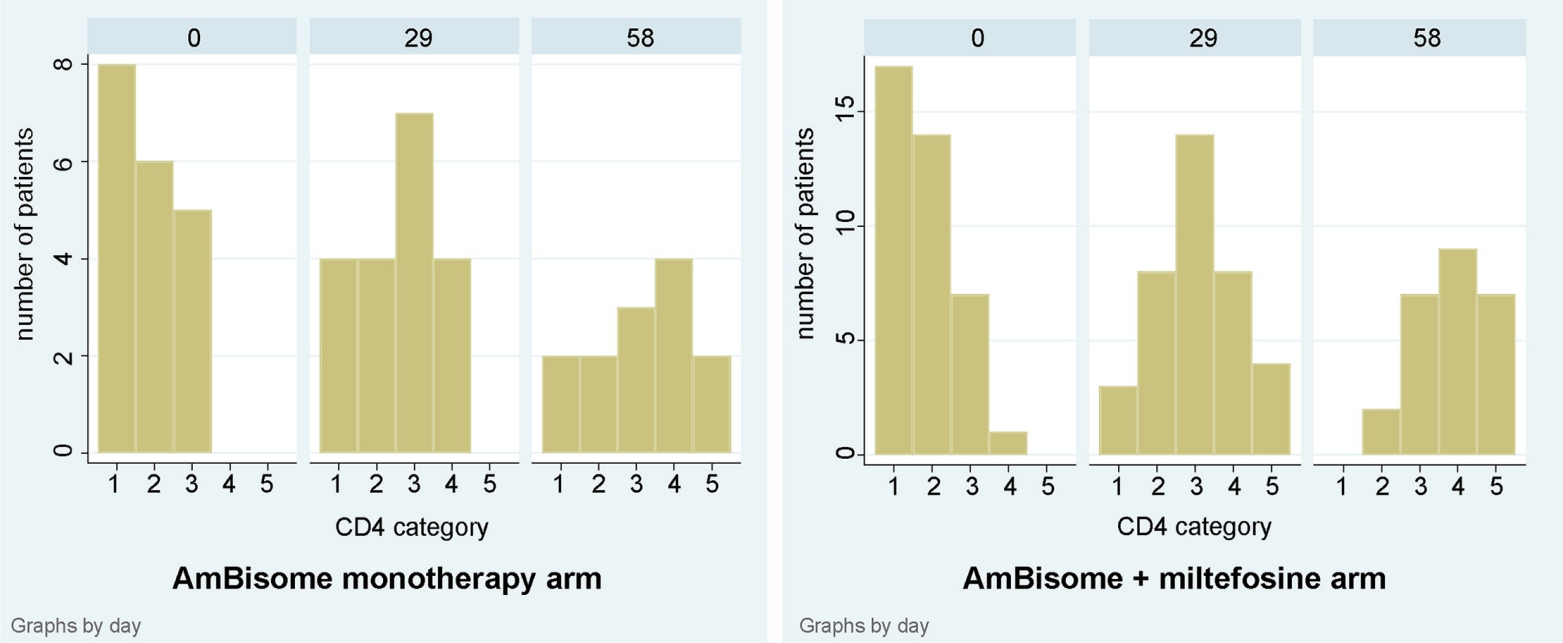
CD4-cell counts at baseline (D0), D29 and D58. Categories are Cat 1 = <50, Cat 2 = 50–99, Cat 3 = 100–199, Cat 4 = 200–349, Cat 5 = ≥350 cells/μl. (A) AmBisome monotherapy arm (B) AmBisome+miltefosine combination arm.

At admission, approximately 70% of patients were on antiretroviral treatment; overall and by arm. The tenofovir-lamivudine-efavirenz combination, the first line treatment according to Ethiopian guidelines, was the most common ARV drug combination used. Only two patients were receiving a protease inhibitor based regimen. All newly diagnosed HIV patients started the ARV treatment after completion of the VL treatment, except for one refusal. Three patients changed their ARV regimen during the VL treatment. CD4 recovery was substantial at the end of VL treatment compared to baseline, 23 patients presented with CD4 count above 200 cells/μl compared to only one at baseline (Fig 4A and 4B). In the monotherapy arm, the average increase in CD4 from baseline to day 29 was 52 cells/μl (95% CI 24–79, paired t-test p = 0·001) and in the combination arm 111 cells/μl (95% CI 67–155 paired t-test p<0·001). HIV viral load was available for 55 patients at baseline (Table 2). Only 16 patients had undetectable viral load among the 41 already receiving ART treatment at inclusion.

## Discussion

The burden of VL in HIV patients and the associated poor treatment outcome with all currently available medicines underline the urgent need for better treatment approaches. This trial was therefore designed to evaluate a combination treatment (AmBisome plus miltefosine) and the current WHO recommended regimen (AmBisome monotherapy), with the aim of providing better quality evidence for future guidelines. The combination arm showed an efficacy rate of 67% on day 29 and 88% on day 58 with prolonged treatment (ITT). In the PP, efficacy at D58 reached 91%.

In 2008, Ter Horst and colleagues [16] made 5 recommendations for the management of VL and HIV co-infected patients in Ethiopia: (i) ART should be provided to all HIV positive individuals; (ii) VL should be an AIDS-defining illness and a valid entry point to ART, irrespective of CD4 count, to reduce the chance of relapse; (iii) secondary prophylaxis is necessary when the risk of relapse is high; (iv) parasitological clearance is a crucial end point for VL treatment; and (v) combination therapy can minimize the risk of developing resistance. Our study focuses on an initial effective treatment defined by parasite clearance at the end of VL therapy (responding to points iv and v), supplemented by secondary prophylaxis and ART (responding to points ii and iii; manuscript in preparation).

The limited number of study reports available on HIV-VL treatment in the region generally show poor treatment outcomes with all the available anti-leishmanial drugs. In a previous study that compared SSG and miltefosine for the treatment of VL, a subgroup analysis showed cure rates of 90% and 78%, and death rates of 7% and 2% for SSG and miltefosine respectively [31]. This study included mainly primary VL, and a death rate of 19% was also reported among patients with unknown HIV status and treated with SSG (patients suspected of advanced AIDS disease). Subsequent studies evaluating SSG in treatment of HIV-VL co-infected patients showed a cure rate that ranged from 43% to 70%, and death rates during treatment that ranged from 14–17% (or ~15%) [2]. AmBisome at a 30 mg/kg dose showed a cure rate of 60% and failure rate of 32%, with a worse outcome in relapsed cases as compared to primary HIV-VL co-infected patients [12].

A randomized trial that enrolled 57 HIV-VL patients from Spain, infected with a different species (*Leishmania infantum*), compared two regimens of amphotericin B lipid complex (ABLC 15 mg/kg and 30 mg/kg total dose), with meglumine antimoniate [32]. The initial cure rates were 33% (95% CI 13%-59%), 42% (95% CI 16%-62%), and 37% (95% CI 16%-62%), respectively. Treatment with the amphotericin B lipid formulation had fewer safety concerns and treatment discontinuations. Despite the limited number of patients per arm in this randomized study (18, 20, and 19 respectively), this evidence, together with a number of reported case series using AmBisome, was used by the WHO Expert Committee to recommend the use of amphotericin B lipid formulations up to 40mg/kg for the treatment of HIV-VL co-infected patients with an ‘A’ grade of evidence [10].

The clinical trial described here is the first to evaluate the efficacy of the combined regimen of AmBisome and 28-day miltefosine. It has demonstrated much higher efficacy in African HIV-VL co-infected patients than the previous trial in Spain using ABLC monotherapy [32]. This result was obtained when the combination was used within a strategy of extended treatment based on the patient’s initial clinical and parasitological response to treatment. Indeed, this strategy achieved a parasite clearance rate of 91% at the end of treatment for patients with high compliance (i.e. *per protocol* analysis population). Notably, the combination treatment offers encouraging efficacy in relapse patients with previous history of VL treatment, as they represent half of the patient population included in the trial.

Importantly, in the context of this trial, we observed the value of parasitological assessment at the end of treatment, especially for HIV-VL patients, clinical evaluation not being sufficiently sensitive to detect patients who failed to clear parasites after the initial treatment. This allowed us to demonstrate that prolongation of same treatment can be effective.

In terms of safety, although all patients experienced at least one adverse event, which is to be expected in this seriously ill population, no AE led to VL treatment discontinuation. Adverse drug reactions were reported in a significant number of patients, but most events were of mild intensity, and mostly corresponded to a single episode of vomiting, known to be associated with miltefosine. Sporadic hypokalaemia remains a concern with AmBisome and requires close monitoring, as previously reported in India [33]. These data therefore suggest a satisfactory safety profile in a population with a high burden of concomitant illness and medication. Prolonging treatment using the extended treatment strategy does not seem to create any safety concern impacting compliance to treatment, although the number of patients in the trial was not conducive to detecting rare events.

Although the combination regimen and treatment strategy identified by this randomized trial shows promising results for the HIV-VL co-infected population, the medicines used remain very expensive, and quality of HIV care remains a challenge to ensuring long-term patient survival. VL control programs in the region are mostly dependent on the support of international organizations that should consider HIV-VL patients as fully part of their main target, despite the financial burden that they represent. Indeed, since HIV-VL co-infected patients are chronically infected, frequently suffer relapse episodes (with high parasitaemia), and can infect sand flies [15], they act as a reservoir in the population. Implementation of a regimen that enhances parasite clearance is thus of major importance both for the individual patient and at the public health level to reduce circulation of the parasite in the community. Ensuring access and optimal care for these patients could impact the efficacy of elimination programmes such as the Indian Kala-azar Elimination Program.

Considering both individual and public health benefits, there is a strong case for the prompt adoption of this strategy into national and regional African guidelines. The Ethiopian authorities have committed to rapidly endorsing the AmBisome and miltefosine combination as the first line regimen in their national treatment guidelines for HIV-VL co-infected patients.

Because both arms crossed the triangular boundary on the lower side, corresponding to a lack of promise, defined as an efficacy of less than 90%, this can be considered as a limitation to the study. This 90% parameter was arguably set too high for this patient population, because efficacy values, for example, in the range 80–90% are still higher than observed in previous studies in HIV-VL patients. Moreover, the data over ran the triangular boundary by a large amount, while the primary analysis assumes this to be negligible. It was therefore felt necessary to take this into account in an additional analysis which is given prominence in the current report. Because of the scarcity of suitable clinical trial sites accessible to patients from remote areas and providing sufficient level of security and stability, the trial was not designed or powered for a comparison of efficacy between arms. However, there is no overlap between 95% CIs around the ITT efficacy at D58 accounting for over-run, tending to suggest higher D58 efficacy in the combination arm.

The numbers of HIV-VL co-infected patients seems to be decreasing in Ethiopia, probably due to the implementation of prevention strategies and large scale anti-retroviral treatment programs. However, some gaps remain in identifying patients with low compliance to HIV treatment, leading to ART failure and emergence of viral resistance, with a consequently late switch to second line ARV treatment. HIV viral load services are limited and results are reported only after long delays. Suboptimal HIV treatment might result in re-emergence of VL cases. This study was not designed to evaluate the importance of ART regimen on the efficacy and sustainability of the response to VL treatment. Closer monitoring of the evolution of CD4, cytokine profile, and HIV viral load would have allowed for a better understanding of the pathophysiology of co-infection, but this was not possible in the clinical settings where the trial was conducted.

In terms of generalizability, geographic variation in efficacy is observed with the commonly used anti-leishmanial medicines, in particular between Africa and India. This study was conducted in Northwest Ethiopia where, as in Sudan, *L*. *donovani* has high strain diversity and is often difficult to treat. These results, even if encouraging, cannot be extrapolated to other settings without reservation. Experience shows that in the Indian subcontinent patients usually respond to lower doses of treatment compared to in Africa. A retrospective study conducted in India with a similar combination regimen (same dose of AmBisome (30 mg/kg) but 14 instead of 28 days of miltefosine) suggested this regimen would be safe, effective, and tolerable [34]. There is now an ongoing randomized trial in India to evaluate this regimen (CTRI/2015/05/005807). In Europe and Brazil, which share the same parasite *L*. *infantum*, differences in the circulating parasites would probably justify bridging studies.

In conclusion, the results of this randomized trial strongly support a change in the treatment recommendations for HIV-VL co-infected patients, from AmBisome monotherapy to combination therapy as the first line treatment. A new case management strategy where duration of treatment is dependent on reaching a negative parasitology, by using one or two rounds of treatment, should be adopted. AmBisome-miltefosine combination therapy has a satisfactory safety profile and is highly efficacious.

## Supporting information

S1 Protocol Study protocol version 2.0 dated 29 Nov 2012. (PDF)

S1 Approval Initial approval by the Gondar University Ethics Committee. (PDF)

S2 Approval Initial approval by the National Research Ethics Review Committee. (PDF)

S1 CONSORT CONSORT checklist. (PDF)

S1 Table Rescue medication. (DOCX)
