# Supplementary material for: A randomized trial of AmBisome monotherapy and AmBisome and miltefosine combination to treat visceral leishmaniasis in HIV co-infected patients in Ethiopia
Source: PLoS Negl Trop Dis. 2019 Jan 17;13(1):e0006988. doi: 10.1371/journal.pntd.0006988 (PMC6336227; doi:10.1371/journal.pntd.0006988)
Supplement: S1 Protocol — (PDF) [file pntd.0006988.s001.pdf]

CLINICAL TRIAL PROTOCOL

A randomized trial of AmBisome® monotherapy and combination of AmBisome® and miltefosine for the treatment of VL in HIV positive patients in Ethiopia followed by secondary VL prophylactic treatment with pentamidine

**Name of product(s)/ Project code**	Liposomal amphotericin B (AmBisome®)<br>Miltefosine (Impavido®)
**Drug Class**	Polyene, alkylphosphocholine
**Phase**	Phase III
**Indication**	HIV and VL co-infected patients
**Protocol Number**	**HIV/VL 0511**
**Sponsor**	DNDi, Chemin Louis Dunant, 15, 1202 GENEVA, Switzerland<br>Phone: +41 22 906 9230
**Co-sponsor**	MSF Operational Centre Amsterdam

Coordinating Investigator/Principal Investigator	**Principal Investigators**<br>❖ Coordinating Investigator: Prof Asrat Hailu<br>❖ Gondar site investigator: Dr Ermias Diro<br>❖ Abdurafi site investigator: Dr. Stille Kolja , Médecins Sans Frontières (MSF)<br><br>**Co-investigators**<br>❖ Dr Koert Ritmeijer, Médecins Sans Frontières, Amsterdam<br>❖ Dr Sisay Yifru, College of Health Sciences, Gondar University<br>❖ Dr Johan van Griensven, Institute of Tropical Medicine, Antwerp<br>❖ Prof Ed Zijlstra, DNDi<br>❖ Dr. Thomas Dorlo, Slotervaart Hospital, Amsterdam<br><br>**Project management:**<br>❖ Nathalie Strub-Wourgaft, DNDi<br>❖ Clélia Bardonneau, DNDi<br>❖ Sally Ellis, DNDi<br><br>**Study statisticians:**<br>❖ Neal Alexander, London School of Hygiene and Tropical Medicine<br>❖ Tansy Edwards, London School of Hygiene and Tropical Medicine
**Protocol Version / Date**	FINAL Version / 29th November 2012 / 2nd Version

The information contained in this document is confidential. It is to be used by investigators, potential investigators, consultants, or applicable independent ethics committees. It is understood that this information will not be disclosed to others without written authorisation from DNDi, except where required by applicable local laws

SIGNATURES

DNDi Signature Page

Medically qualified DNDi Officer

Signature ________________________ ________________
Date of Signature (DD/mmm/YY)

Name	Nathalie Strub-Wourgaft
Title	Medical Director
Institution	DNDi
Address	15 Chemin Louis-Dunant, 1202 Genève, Switzerland

Statistician

Signature ________________________ ________________
Date of Signature (DD/mmm/YY)

Name	Neal Alexander
Title	Lead Statistician
Institution	London School of Hygiene & Tropical Medicine
Address	Keppel Street, London WC1E 7HT, UK

Investigators Signature Page

I have read this protocol and agree that it contains all necessary details for carrying out this trial. I will conduct the trial as outlined herein and will complete the trial within the time designated.
I will provide copies of the protocol and all pertinent information to all individuals responsible to me who assist in the conduct of this trial. I will discuss this material with them to ensure they are fully informed regarding the drug and the conduct of the trial.
I will use only the informed consent form approved by the sponsor or its representative and will fulfill all responsibilities for submitting pertinent information to the Institutional Review Board/Independent Ethics Committee (IRB/IEC) responsible for this trial.
I agree that the sponsor or its representatives shall have access to any source documents from which case report form information may have been generated.

Coordinating Investigator

Signature ______________________________ ________________
Date of Signature (DD/MM/YY)

Name ______________________________

Title ______________________________

Institution ______________________________

Country ______________________________

Principal Investigator

Signature ______________________________ ________________
Date of Signature (DD/MM/YY)

Name ______________________________

Title ______________________________

Institution ______________________________

Country ______________________________

TABLE OF CONTENTS

TABLE OF CONTENTS 5
ABBREVIATIONS – GLOSSARY OF TERMS 7
SYNOPSIS 10
1. BACKGROUND AND STUDY RATIONALE 17
2. STUDY OBJECTIVES AND ENDPOINTS 23
2.1 General Objectives 23
2.1.1. Primary Objective 24
2.1.2. Secondary Objectives 24
2.1.3. Other Objectives 24
2.2 Study Endpoints 24
2.2.1 Primary Endpoint 24
2.2.2 Secondary Endpoint(s) 24
3. STUDY DESIGN AND STUDY DESIGN RATIONALE 25
3.1. Study design 25
3.2. Study duration and duration of subject participation 25
3.3. Rationale of study design 25
4. SELECTION OF SUBJECTS 28
4.1. Inclusion criteria 28
4.2. Exclusion criteria 28
5. SCHEDULE OF EVENTS 30
6. ENROLMENT PROCEDURES 31
7. TREATMENTS 32
7.1. Investigational Product 32
7.2. Doses and treatment regimens 33
7.3. Drugs labelling, packaging 33
7.4. Accountability 33
7.5. Storage 33
7.6. Blinding and procedures for unblinding 34
7.7. Concomitant medications 34
7.8. Secondary prophylaxis 34
7.9. Rescue medication 34
8. STUDY ASSESSMENTS 35
8.1. Timing of Assessments 35
8.2. Baseline Assessments 35
8.3. Assessment of Efficacy 35
8.4. Assessment of Safety 36
8.5. Follow up assessments 37
8.6. Other assessments 37
8.7. Adverse event definitions and reporting 43

9. WITHDRAWAL CRITERIA 46
9.1. Rules in case of treatment suspension or interruption 46
9.2. Rules for permanently interrupting study treatment 46
10. DATA ANALYSIS AND STATISTICAL METHODS 47
10.1. Sample size determination 47
10.2. Definition of study populations included in the analysis 47
10.3. Subject Disposition 48
10.4. Baseline 48
10.5. Treatment Compliance 48
10.6. Efficacy Analysis 48
10.7. Safety Analysis 49
10.8. Analysis of other endpoints 49
10.9. Interim analysis 49
11. DATA SAFETY MONITORING BOARD 50
12. QUALITY ASSURANCE AND QUALITY CONTROL PROCEDURES 50
12.1. Investigator's file 50
12.2. Case report forms (CRFs) 50
12.3. Source documents 51
12.4. Record Retention 51
12.5. Monitoring 51
12.6. Audits and inspections 52
12.7. Data Management 52
12.8. Confidentiality of trial documents and subjects records 52
13. PROTOCOL AMENDMENTS 52
14. TERMINATION OF THE STUDY 53
15. ETHICS 54
15.1. Informed consent process 54
15.2. Ethical aspects of subject inclusion and study procedures 55
15.3. Ethical aspects of study treatments 56
15.4 Patient costs 56
16 INSURANCE AND LIABILITY 56
17 REPORTING AND PUBLICATION 57
ANY REPORTS INCLUDING STUDY RESULTS WILL BE PROVIDED TO THE ETHICAL AND REGULATORY BODIES ACCORDING TO LOCAL REQUIREMENTS 57
REFERENCES 58

ABBREVIATIONS – GLOSSARY OF TERMS

ABLC	Amphotericin B Lipid Complex
AE	Adverse Event
ALT	Alanine aminotransferase (SGPT)
AMB	Amphotericin B
AP	Alkaline Phosphatase
ART	Antiretroviral Treatment
AST	Aspartate aminotransferase (SGOT)
AZT	Zidovudine
CL	Cuteanous Leishmaniasis
CPT	Cotrimoxazole Preventive Therapy
CRF	Case report form
DBS	Dried Blood Spot
DNDi	Drugs for Neglected Diseases *initiative*
DSMB	Data Safety Monitoring Board
EDTA	Ethylenediaminetetraacetic Acid
EFV	Efavirenz
FDA	Food and Drug Administration
FBC	Full Blood Count
GCP	Good clinical practice
HAART	Highly Active Anti-Retroviral Therapy
HIV	Human Immunodeficiency Virus
IEC	Independent ethics committee
ICH	International Conferences on Harmonization
IM	Intramuscular
ITM	Institute of Tropical Medecine
ITT	Intention to treat
IUCD	Intrauterine contraceptive device
IV	Intravenous
L-AMB	Liposomal Amphotericin B (Ambisome®)
LEAP	Leishmaniasis East Africa Platform
LPV	Lopinavir
LTFU	Lost to Follow up
N/A	Not available
MF	Miltefosine
mRNA	Messenger Ribonucleic Acid
MSF	Médecins Sans Frontières
NNRTI	Non-nucleoside Reverse Transcriptase Inhibitor
NVP	Nevirapine
P-gp	P-glycoprotein
PI	Principal investigator
PI	Protease Inhibitor
PK	Pharmacokinetics

PKDL	Post-kala-azar Dermal Leishmaniasis
PP	Per protocol
RBC	Red blood count
RTV	Ritonavir
SAE	Serious Adverse Event
SbV	Stibogluconate (pentavalent)
SOP	Standard Operating Procedure
SSG	Sodium Stibogluconate
TOC	Test of Cure
ULN	Upper Limit of Normal
VL	Visceral Leishmaniasis
VCT	Voluntary Counselling and Testing
WBC	White blood count
WHO	World Health Organization
WNL	Within Normal Limits
3TC	Lamivudine

SYNOPSIS

**Background Information and Trial Rationale**	Co-infection of human immunodeficiency virus (HIV) and visceral leishmaniasis (VL) was described extensively in the Mediterranean area where an increase of VL cases was noted in HIV-infected individuals in the 1980s. The most important features of co-infection include poor outcome, increased drug toxicity and relapse after treatment with the need for maintenance therapy[1]. The risk of death from VL is nine times higher in those who are co-infected with HIV. In addition there is high parasitaemia with increased potential for transmission[2].<br><br>The incidence of cases in the Mediterranean area has decreased with the introduction of highly active anti-retroviral therapy (HAART); however, with further spread of HIV in VL endemic areas, an increase in co-infected cases has been reported in Africa (Ethiopia) and Latin America (Brazil).<br><br>There are few treatment studies in co-infected patients. In the Mediterranean area, high dose AmBisome® (40 mg/kg total dose) is considered the best treatment option and this is the current formal WHO recommendation.[3]<br><br>According to the Ethiopian National Guidelines (2006), Sodium Stibogluconate (SSG) and amphotericin B deoxycholate are the first and second line treatments for VL respectively. There are no specific recommendations for HIV-VL co-infected patients. Studies from Ethiopia in co-infected patients however have shown that treatment with SSG has considerable toxicity (mainly pancreatitis) with increased risk of death (odds ratio=6)[4] . Miltefosine is better tolerated but has a low cure rate (60% at 6 months). AmBisome® is not routinely available except in special programmes.<br><br>Recent experience of Médecins Sans Frontières (MSF) in Ethiopia (Abdurafi centre) shows initial cure rates of 74% with AmBisome® 30 mg/kg total dose in 116 patients with a first episode of VL, and only 60% overall in first episode and relapse patients (n=195) of VL with HIV co-infection[5]. Compassionate management of such difficult cases has also included combination treatment of AmBisome® (30 mg/kg total dose) with miltefosine (2.5 mg/kg daily for 28 days) with promising initial results.<br><br>It should be noted that a preclinical study (unpublished) has also been conducted by DNDi where no major safety interactions were noted between the combination of AmBisome® and miltefosine. Activity enhancement (synergy) has also been noted between the two drugs in another preclinical study.[6] The combination has also been used successfully to treat immunocompetent VL patients in lower doses in phase II and III studies in India.[7]<br><br>There is little information from other endemic areas; experience from a number of centres in the state of São Paulo, Brazil seem to give similar low cure rates with AmBisome® monotherapy at 21 mg/kg total dose in co-infected patients.<br><br>This protocol will evaluate the efficacy and safety of the combination of AmBisome® 30 mg/kg with miltefosine (2.5 mg/kg for 28 days) and AmBisome® monotherapy (high dose: 40 mg/kg) in Ethiopia.

**Trial Objectives**	**General Objectives**<br>The overall objective of this trial is to identify a safe and effective treatment for VL in HIV co-infected patients.<br><br>**Primary Objective:**<br>To evaluate at day 29 assessment the efficacy of a combination regimen of AmBisome® + miltefosine and AmBisome® monotherapy in Ethiopian co-infected HIV + VL patients.<br><br>**Secondary Objectives:**<br>1. To evaluate relapse-free survival at day 390 (after initial cure at day 29 or cure at day 58 after extended treatment).<br>2. To assess safety of the regimens.<br><br>**Other objectives:**<br>1.To evaluate of viral load and CD4 count in all patients<br>2. To evaluate the pharmacokinetics of ARV, Ambisome and miltefosine and immune function markers in a subset of patients
**Trial Endpoints**	**Primary Efficacy Endpoints:**<br>The primary endpoint is initial parasitological cure at day 29 and is defined as absence of parasites in tissue aspirate at day 29.<br><br>**Secondary Endpoint:**<br>**Relapse-free survival at 12 months as defined as**<br>• The patient being alive and disease-free from day 29 (if initially cured) or day 58 (for those who received an extended treatment) and remains disease-free until the last follow-up assessment (i.e. day 390).<br><br>**Safety**<br>• Assessment of safety during treatment and follow-up based on clinical adverse events, laboratory parameters during treatment and 1 month (after end of initial treatment or end of extended treatment) follow-up.
**Trial Design**	A randomized, parallel arm, open-label clinical trial to assess the safety and efficacy of the combination of AmBisome® plus miltefosine and AmBisome® alone for the treatment of VL in HIV co-infected patients in Ethiopia.

**Main Entry Criteria**<br>Inclusion<br>Exclusion	**Inclusion criteria**:<br>• Confirmed HIV positive test (2 rapid diagnostics tests (RDTs) followed by a confirmatory ELISA test).<br>• Diagnosis of VL (first episode or relapse) confirmed by bone marrow or spleen aspirate.*<br>• Male and female age: 18-60 years.<br>• Written informed consent from the patient.**<br><br>**Exclusion criteria**<br>The presence of any of the following will exclude a patient from study enrolment:<br>• Women of child-bearing potential (defined as women who have achieved menarche) who are not using an assured method of contraception or are unwilling to use an assured method of contraception for the duration of treatment and four months after.***<br>• Pregnant women or breast-feeding mothers.<br>• Patients with grade 2 or 3 post kala-azar dermal leishmaniasis (PKDL) lesions.<br>• Clinical or biological evidence of severe cardiac, renal or hepatic impairment.<br>• Known hypersensitivity to AmBisome® and/or miltefosine.<br>• Patients receiving allopurinol treatment<br><br>** Patients with a platelet count less than 40,000/ µL or Hemoglobin (Hb) less than 5g/dL, without palpable spleen or with bleeding tendency (e.g. epistaxis) should be diagnosed by bone marrow rather than spleen aspirate. Ideally, the same type of tissue aspirate should be performed at screening and at day 29 assessment (except if not clinically indicated). During follow up, a spleen aspirate can be performed, if required, provided there is recovery of the platelet count, Hemoglobin and/or bleeding tendency.*<br><br>*** If the patient is illiterate an impartial witness should be present during the consenting procedure and should also sign.*<br><br>**** Women who are sexually active and not using an assured method of contraception i.e. either IUCD or medroxyprogesterone acetate (Depo-Provera) but who wish to participate in the trial must sign an additional consent form and agree to receive a long-acting assured form of contraception. Oral contraceptives are not considered adequate in this context because of the high prevalence of vomiting and diarrhoea associated with miltefosine treatment. As these patients are HIV positive, the above contraceptive methods would be in addition to the use of condoms which should be normal practice and will be encouraged throughout the study.*

**Study Duration**	Treatment duration will be 28 days or 56 days in case of extended treatment (please refer to point b).<br><br>a) If at day 29 assessment, tissue aspirate is parasite negative, the patient will be eligible for secondary prophylaxis* and enter the of one-year follow-up phase (i.e. day 58, 210 and 390 assessments) following initial treatment.<br><br>b) If at day 29 assessment, tissue aspirate is parasite positive and the patient is well (i.e. no fever and clinical improvement of VL signs and symptoms compared to baseline), s/he will receive another complete course of the same treatment. (Note: these patients will still be considered treatment failures at the primary endpoint).<br><br>c) If at day 29 assessment, tissue aspirate is positive and the patient is unwell, s/he will also be considered a treatment failure, but the patient will be treated with a rescue therapy.<br><br>Patients who have an extended treatment (option b) will be evaluated on day 58. Those who still have a parasite positive tissue aspirate at this assessment will be offered a rescue treatment. Those who are negative at this assessment will be evaluated a month later (i.e. day 86) to assess their eligibility to start secondary prophylaxis. *<br>Patients who have rescue treatment (option c) will be evaluated on day 58. Those who still have a parasite positive tissue aspirate at this assessment may be offered another rescue treatment. Those who are negative will be offered secondary prophylaxis, if eligible. *<br>All patients, independently of their outcome at day 29 and/or day 58, will enter the follow-up assessments (i.e. day 210 and 390 assessments).<br><br>All patients who are not yet on antiretroviral treatment (ART) will commence ART once they have completed the routine VCT procedures. This will be approximately 2-3 weeks after commencement of VL treatment. Those who are still critically ill according to clinical judgment of the attending physician will have ART deferred until clinical improvement. Patients who are already on ART at diagnosis of VL will continue ART throughout the study.<br>The ART treatment will be provided to the patient through the Ethiopian ART programme before (if applicable), during and after the study.<br><br>** Secondary prophylaxis given will be pentamidine 4mg/kg IM once a month. Patients will be eligible for this if their CD4 count is < 200 cells/mL and there is no contraindication (e.g. renal failure, diabetes mellitus, known hypersensitivity) for the drug used. Secondary prophylaxis will normally be continued until the patient has sufficient CD4 recovery (over 200 cells/mL at day 390 assessment). However, it would be stopped in case of relapse, adverse events requiring discontinuation of medication or if the patient requests to stop the prophylaxis.*

<table>
<tr><td>Test Drugs</td><td>
AmBisome®
This is a liposomal formulation of amphotericin B deoxycholate manufactured and supplied by Gilead Sciences, USA. Each box of AmBisome® is supplied as a pack of 10 vials each containing 50 mg of lyophilised AmBisome®.
AmBisome® must be used according to the manufacturer's instructions.

Dosing schedule:

<u>AmBisome® monotherapy</u>:

• Adapted from WHO recommendations for co-infected patients:
40 mg/kg total dose: IV infusion of 5 mg/kg on day 1-5, 10, 17, 24.

<u>AmBisome® in combination with Miltefosine</u>:

• AmBisome®: 30 mg/kg total dose: IV infusion 5 mg/kg on day 1, 3, 5, 7, 9, 11.

• Miltefosine: every day during 28 days.
The dose is calculated according to the patient weight:
  • ≤ 25 Kg, the patient receives 50mg (i.e. 1 x 50mg capsule) per day.
  • > 25 Kg, the patient receives 100mg (i.e. 2 x 50mg capsules) per day.
</td></tr>
</table>

**Statistics**<br><br>Summary of analysis	**Primary Endpoint Analysis: Day 29**<br>The study is designed and will be analysed according to group-sequential methods, specifically the triangular test. The triangular test is one way of analysing group sequential trials and uses straight line stopping boundaries. It involves analysing the data as they accumulate, with points being plotted relative to a triangular region and stopping when the upper or lower boundary of the region is crossed (Graph 1). It has the advantage over other methods of analysing group sequential trials, such as the discrete sequential probability ratio test, in that a closed continuation region is used ensuring that the trial stops within a pre-specified maximum sample size.[8,9]<br>The type I error rate and power of the study were set to 5% and 95%, respectively (α=0.05, β =0.05) with parameters p_0=0.75 and p_a=0.90. Based on these specifications, the boundaries of the test were calculated for the null hypothesis, H_0:$p \le p_0$, and the alternative hypothesis, H_a: $p > p_0$ (see Graph 1). Based on the rationale above, rejecting the null hypothesis, H_0:$p \le p_0$, (crossing the upper boundary) means that we can consider the regimen for compassionate use while failing to reject the null hypothesis (crossing the lower boundary) means that we cannot consider the regimen for compassionate use. Data from each arm will be analysed (for the evaluable patient population or per protocol analysis) after every 10 patients reach the primary endpoint of initial cure, where the actual values of *Z* and *V* will be calculated and then plotted on the graph. Depending on where the point falls, that arm of the trial will either continue collecting data for another 10 patients or will stop, concluding with a rejection of the null hypothesis (adequate efficacy) or a non-rejection of the null hypothesis (inadequate efficacy). In the final analysis, asymptotic properties will be used to calculate unbiased estimators of the response rate and its 95% confidence interval taking into account the sequential nature of the analyses.[10]<br><br>**Secondary Endpoint Analysis:**<br>When the analyses concludes that an arm should be stopped (i.e. a boundary has been crossed), survival at 1 year will also be evaluated. Due to sequential design employed and the different treatment options to be determined by day 29 outcomes (i.e. repeat treatment and/or pentamidine prophylaxis), subsequent analyses (i.e. the secondary endpoint) are likely to be descriptive only. Such descriptive analyses will focus on survival (alive and disease-free) at 12 months post day 29 assessment and relapses between day 29 and 12 months follow-up, by arm and day 29 outcome.<br><br>**Safety Analysis**<br>Adverse events (AEs) will be described on treatment and during follow-up. AEs will be coded using MedDRA and tabulated by severity / CTC V4 AE grading. Causality of AEs will also be tabulated. Serious Adverse Events (SAEs) will be described by individual narratives based on the SAE reports provided by the site investigators. Summary statistics will be tabulated for haematological and biochemical parameters as well as for ART efficacy (CD4 count, viral load) at each time point.
Randomization	Patients will be randomized and will be stratified by centre (*i.e.* Abdurafi and Gondar) and by whether or not the current VL episode is the first (*i.e.* primary and relapses cases). Randomisation codes will be prepared by the DNDi /LEAP data centre and provided to the centres in sealed opaque envelopes and will be under the control of the site principal investigator.
Sample size	The sample size will be determined according to the sequential design for the primary outcome to be measured at day 29.<br>Since we are using a sequential approach, the sample size is not defined or known in advance. The maximum sample size needed per arm would be 66 patients (132 total patients) and the maximum analyses performed would be 7 (see Graph 1, upper horizontal axis). The actual sample size may be less than this, with its value depending on the value of the proportion cured.
Early stopping rule for safety	The study design will also account for lack of efficacy /rescue treatment given for early cessation of study arm.

	Graph 1: Triangular region for study arms showing the boundaries for analysing the sequential trial using the Triangular Test, with the following parameters (p_0 =0.75, p_a=0.9, α=0.05, β =0.05 and n=10). 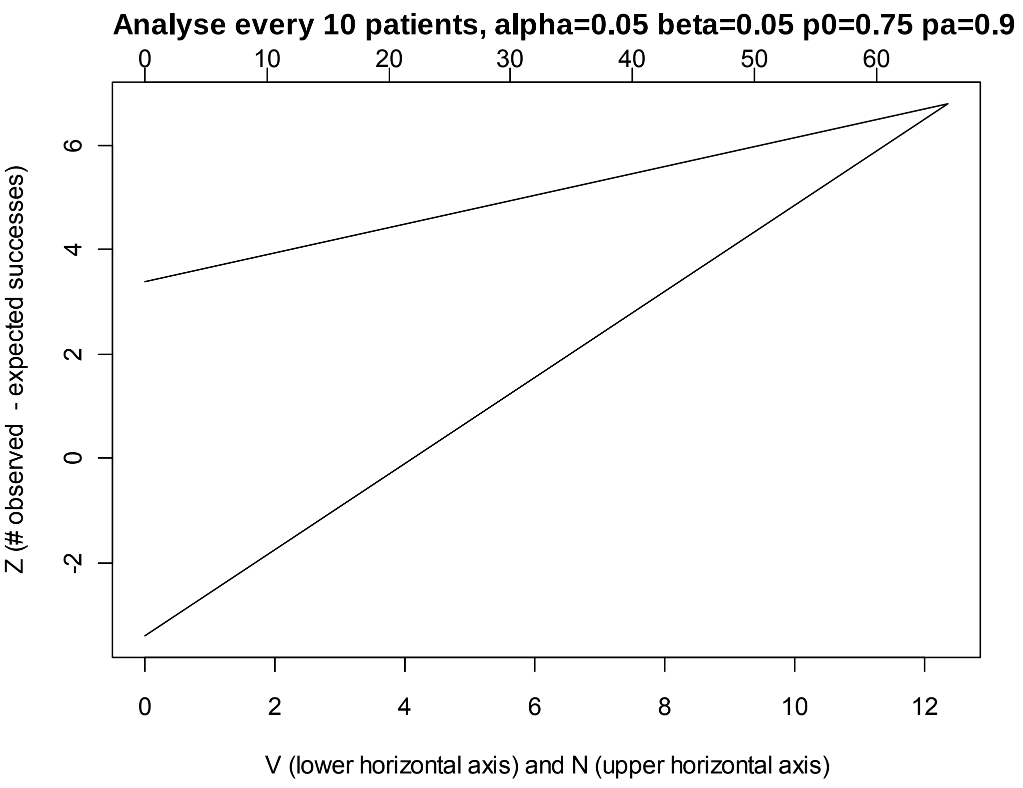

1. Background and Study Rationale

Background

Epidemiology

In 2009, 33 million people were reported to be living with HIV/AIDS with 2.6 million of which were newly infected; 69% of these new infections occured in sub-Saharan Africa. Leishmaniasis is recognized as one of the most neglected diseases with 12 million people infected and 2 million new infections annually. Of these 500,000 are with visceral leishmaniasis. The two epidemics geographically overlap and the first cases of co-infection were reported from southern Europe in 1985. Since then, a considerable amount of knowledge and experience has been accumulated that can be summarized as follows:

HIV and VL mutually influence each other as they both affect cellular immunity; as a consequence, the risk of VL may be up to 2300 times higher in an HIV-infected individual. Conversely, concurrent VL accelerates HIV progression[11].

VL occurs in co-infected patients when they have already developed advanced HIV disease (AIDS); in virtually all patients CD4 count at presentation are below 200 cells/mL.

Co-infected patients have ample parasites circulating in the peripheral blood that contribute to transmission by the sandfly vector. In the Mediterranean most co-infected cases were seen in Spain among IV drug abusers; a new transmission cycle was found by means of the sharing of used needles without the need for the sandfly vector.

While the clinical presentation is not essentially different, co-infected patients may also have atypical presentations and clinical suspicion of VL may be decreased as in HIV/AIDS patients classical clinical signs and features such as splenomegaly and pancytopenia may be caused by other HIV-related pathology or medication.

While parasitological diagnosis is on average more sensitive in co-infected patients, serodiagnosis is cumbersome because of poor humoral antibody responses. This is of concern as many patients relapse and may be reluctant to undergo repeated invasive diagnostic procedures such as bone marrow aspiration.

The most important problem is treatment; when compared with VL patients who are not infected with HIV, cure rates for HIV co-infected patients are lower, drug toxicity is increased and mortality rates are higher. Relapses are common; studies have shown that most if not all are true relapses with the same leishmania strain although some new infections may also occur. Persistent low CD4 counts may play a role and with each relapse the response to treatment is lower[12].

The incidence of cases in the Mediterranean area has decreased with the introduction of anti-retroviral therapy (ART); it is probable that ART prevents asymptomatic patients from becoming symptomatic by restoring immunity.

While the epidemic of co-infection has decreased in the Mediterranean area, with further spread of HIV in VL endemic areas, an increase in co-infected cases has been reported in Africa (Ethiopia)[13] and in Latin-America (Brazil)[14].

In Ethiopia the national HIV prevalence is 2.1% (MoH, 2007). VL is reported from at

least 40 areas but 60% of cases occur in the Metema and Humera lowlands[15]. Co-infection rates range from 18-31%[16, 17]. Most patients are young men who are among the 300 - 500,000 migrant workers who come each year from the highlands to work in the agricultural schemes; while some are already infected on arrival, it is assumed that others contract HIV by visiting local sex workers.

In Brazil, the number of VL cases is increasing country-wide as is the number of co-infected patients. This is thought to be related to the increasing urbanization[18].

Treatment options for visceral leishmaniasis

Current treatment options and their limitations are summarized in Table 1.

Table 1: Current treatment options for patients with visceral leishmaniasis

Drugs	Limitations
**Pentavalent antimonials**	toxicity (pancreatitis, hepatitis and cardiotoxicity: arrhythmias)<br>30 day iv/im treatment in hospital<br>painful injections
**Amphotericin B**	Needs slow IV infusion; infusion reactions (fever); nephrotoxic; needs lab monitoring
**Liposomal amphotericin B**	expensive<br>requires slow iv infusion over 1-2 hours however long term hospitalization is not required
**Miltefosine**	teratogenic<br>expensive<br>GI toxicity, hepato- & renal toxicity
**Paromomycin**	an aminoglycoside, therefore nephro- and ototoxicity possible: although reversible high tone audiometric shift may occasionally occur during treatment (Sundar, et al. 2007); geographical variation in response (Hailu, 2010)
**Pentamidine**	IM injection<br>Risk of developing diabetes mellitus

In recent years, there has been increasing interest in developing combination regimens that offer the benefit of short duration therapy with reduced toxicity and cost, while maintaining or improving efficacy. In East Africa, a randomized, comparative study of SSG monotherapy (20 mg/kg for 30 days), paromomycin sulphate (PM) monotherapy (20 mg/kg for 21 days) and combination treatment

consisting of PM at 15 mg/kg/day + SSG at 20 mg/kg/day for 17 days (LEAP 0104B) has now been completed and will be reported soon.
Currently a phase II study is being conducted in non-co-infected VL patients that looks at the combination of AmBisome (single dose 10 mg/kg) + SSG 20 mg/kg for 10 days, AmBisome (single dose 10 mg/kg) + miltefosine (2.5 mg/kg for 28 days) and miltefosine monotherapy (2.5 mg/kg for 28 days) (LEAP 0208).

Current knowledge on treatment options for co-infected patients

The treatment of VL patients co-infected with HIV differs considerably from immunocompetent VL patients; the currently available information is summarized below and in Table 2 (studies in the Mediterranean area) and Table 3 (studies in other areas).

1. SSG. A large RCT in Ethiopia has shown an initial efficacy for SSG in co-infected patients of about 90%. However, this figure excludes patients that died during treatment where there was a clinical suspicion that they may have been HIV positive but their HIV status was never confirmed.
   For the comparator arm with miltefosine, the initial cure rate was 78%. All treatment failures in both arms were treated further with SSG; this resulted in end-of –treatment cure rates of 90 and 89%, for those treated initially with SSG and miltefosine, respectively. At 6 months follow-up, relapses were found in 11% and 25%, respectively. SSG caused more severe side-effects such as severe pancreatitis and increased mortality[4]; the risk of death was increased with a factor 6.35 (95% CI 2.5-16.9). In a retrospective study cure rate was lower SbV at 68%[19]. Another study showed that SbV may stimulate HIV replication[20].

2. Paromomycin. There are no data on toxicity and safety in co-infected patients. In HIV negative patients considerable differences in efficacy exist between India and African countries, with unacceptable low cure rates in Sudan[21]. There are concerns about resistance that is readily induced *in vitro* and may occur after prolonged administration in CL patients. Generally it is felt that in Africa PM should only be used in combination with other drugs in particular in Sudan. SSG + PM combination is the current WHO recommended treatment for VL patients in East Africa.

3. Amphotericin B (AMB). This is no longer used in Western countries as safer drugs are now available; it has been widely used in India but is now superseded by miltefosine. In co-infected patients efficacy is equal to that of SbV (initial cure rates 58-82%) with renal toxicity occurring in 18-36%.

4. Liposomal Amphotericin B (Ambisome®). AmBisome is considered safe even also in HIV co-infected patients. It was shown to be effective in Europe in doses of 30-40 mg/kg but it did not prevent relapses. In Ethiopia, cure rates with total dose of 30mg/kg are unacceptably low in HIV co-infected patients. MSF in Ethiopia records a 74% cure rate in primary VL[5]. There is little information from other endemic areas; experience from a number of centres in

the state of São Paulo, Brazil seem to give similar low cure rates with monotherapy AmBisome® at 21 mg/kg total dose in co-infected patients. The official WHO recommendation is 40 mg/kg total dose based on (limited) experience in Europe (table 2).

5. Miltefosine. In a RCT in Ethiopia MF was safer than SSG but efficacy was lower with a relapse rate of 31% (also see above under SSG)[4]. It is potentially teratogenic implying the use of adequate contraceptive cover in women of child-bearing age. Because of the long-half life, this should be maintained for 4 months after treatment when used for 28 days.

6. Pentamidine. There is no information on its use in the primary treatment of VL in co-infected patients; it is mainly used as secondary prophylaxis. It is well-distributed in tissues and slowly excreted. While in previous treatment studies in HIV-negative patients toxicity is well described (mainly development of diabetes mellitus), no serious side-effects are noted when pentamidine is used as secondary prophylaxis.

Table 2: Summary of studies on treatment of HIV-VL co-infected patients in Europe

References	Study drug	Dose	Initial cure rate	Relapse	Side-effects	Study type
Laguna, 1999[22]	SbV	20 mg/kg, 28 days	29/44 (66)	11/24 (46)	Cardiotoxicity in 6; pancreatitis in 3	RCT
	AMB		28/45 (62)	8/24 (46%)	Severe chills in1; venous thrombosis in 1; nephrotox in 16	
Delgado, 1999[23]	SbV	20 mg/kg, 28 days	5/25 (33)	3/15 (20)	Pancreatitis in 5; acute renal failure in 1, leucopenia in 1)	retrospective
Laguna, 2003[24]	SbV	20 mg/kg, 28 days	7/19 (37)	4/7 (57)	Pancreatitis in 4; renal failure in 2;	RCT
	ABLC	3 mg/kg, 5 days	6/18 (33%)	2/4 (50)	Mild infusion-related reactions in 7; renal failure 1	
	ABLC	3 mg/kg, 10 days	8/20 (42)	3/8 (38)	Mild transfusion-related reactions in 8	
Pintado, 2001[25]	SbV	20 mg/kg, 3-4 weeks	61	N/A	Hyperamylasemia in 15.9%; anemia in 14.7%	retrospective
	AMB	0.5-1mg/kg, 3-4 weeks	58	N/A	Anemia in 29.4%; renal failure in 17.6%	
Lopez-Velez, 1998[26]	SbV	20 mg/kg, 28 days	42/51 (82)	N/A	Hyperamylasemia in 8; cardiotoxicity in 9	Retrospective
	AMB	15mg/kg total dose	14/17 (82)	N/A	Anemia in 6; renal failure in 5	
	Penta	4 mg/kg, 28 days	2/3 (67)	N/A	Insulin-dependent diabetes mellitus in 1	
Davidson, 1994[27]	AmBisome®	100 mg for 21 days	11/11 (100)	8/11 (73)	No serious side-effects	Open-label, dose finding
Russo, 1996[28]	AmBisome®	4 mg/kg on days 1 to 5 ,10, 17,24,31 and 38	9/10 (90)	7/7 (100)	No serious side-effects	Open label, dose finding

Table 3. Summary of studies on treatment of HIV-VL co-infected patients in areas other than Europe

Endemic area	References	Drug	Dose	Initial cure	Relapse	Side-effects	Study type
Ethiopia	Berhe, 1999 [29]	SbV	20 mg/kg, for 28 days	17/23 (74)	N/A	N/A	Prospective
Ethiopia	Ritmeijer, 2006 [4]	Milt	100 mg, for 28 days	56/63 (89)	N=52<br>Relapse 16 (31)<br>Deaths 7 (11)<br>Final cure 26 (49)	Vomiting in 41 (serious in 3); diarrhea in 34)	RCT
		SbV	20 mg/kg, 40-60 days	40/44 (90)	N=35<br>Relapse 5 (14)<br>Deaths 5 (11)<br>Final cure 25 (57)	Vomiting in 20 (7 serious); diarrhea in 32	
Ethiopia	Hurissa, 2010 [19]	SbV	20 mg/kg, for 28-30 days	63/92 (68)	13/92 (14)	N/A	Retrospective
India	Sindermann, 2004 [30]	Milt	100 mg, for a mean of 55 days	25/39 (64)	N/A	Vomiting in 10; diarrhea in 4, all mild	Case series
India	Sinha, 2011 [31]	AmBisome®	20-25 mg/kg for 4-15 days	55/55 (100)	8.1% (1 year)<br>26.5% (2 years)	Excellent tolerance	Case series
Ethiopia	Ritmeijer, 2011 [5]	AmBisome®	30mg/kg	74% primary VL<br>38% relapses	N/A	N/A	Case series

Current treatment modalities of VL and VL-HIV co-infected patients in Ethiopia
According to the 2006 Ethiopian National Guidelines, sodium stibogluconate (SSG) and amphotericin B deoxycholate are the first and second line treatments for VL respectively. There are no specific recommendations for HIV-VL co-infected patients. However, these guidelines are currently being revised to recommend that the first line treatment recommended be a combination therapy (SSG + PM) and the second line treatment be Liposomal Amphotericin B (AmBisome®).
Results of available studies are given in Table 3. MSF-Holland was using AmBisome® in a total dose of 30 mg/kg in its Abdurafi treatment centre; initial cure rates have been reported as 74% for primary VL patients and 36% for relapse patients; mortality was 8% and 5% and parasitological failure 16% and 56 %, respectively[5]. Because of these poor responses, compassionate treatment with a combination regimen of Ambisome® 30 mg/kg total dose with miltefosine 2.5 mg/kg for 28 days has been implemented. Preliminary results show an initial cure rate of around 80% (in primary as well as relapse cases)[32].

Rationale

Current experience and published studies (Table 2 & 3) strongly suggest that higher doses and full treatment courses are needed to achieve parasitological cure in HIV co-infected patients in order to prevent relapse. AmBisome® is the safest and most effective drug for the treatment of VL in Asia. However, in non-HIV infected patients in Africa higher doses of AmBisome® are required to obtain similar efficacy[33]; one small phase II clinical trial in Kenya noted that 10 out of 10, 9 out of 10 and only 1 out of 5 patients were cured by total doses of 14mg/kg,10mg/kg and 6mg/kg respectively. In co-infected patients higher doses are recommended.

Based on these data, we proposed to perform a randomized study with two arms:

1. **AmBisome® monotherapy:** 40 mg/kg total dose adapted from the dose recommended by WHO.
2. **Combination of AmBisome® and miltefosine:** Ambisome® in the currently used dose of 30 mg/kg total dose given over 11 days and miltefosine for 28 days.

Based on the study design and because of the unacceptable low efficacy rate of AmBisome® 30 mg/kg monotherapy[5], no comparator arm will be included.

2. Study Objectives and Endpoints

2.1 General Objectives

The overall objective of this trial is to identify a safe and effective treatment for VL in HIV co-infected patients.

2.1.1. Primary Objective

- To evaluate at day 29 assessment the efficacy of a combination regimen of AmBisome® + miltefosine and AmBisome® monotherapy in Ethiopian HIV co- infected VL patients.

2.1.2. Secondary Objectives

- To evaluate the relapse-free survival at day 390 (after initial cure at day 29 or cure at day 58 after extended treatment).
- To assess safety of the regimens (see below)

2.1.3. Other Objectives

On all patients:

- Assessment of response to antiretroviral treatment by measuring CD4 count and HIV viral load during treatment and at day 210 and 390 follow-up assessments.

On a sub-group of patients:

- Pharmacokinetics: Assessment of drugs level to monitor any drug-drug interaction between the proposed VL test drugs and concomitant antiretroviral drugs.
- Assessment of immune responses during treatment and follow-up by measurement of the cytokine profile (direct and by measuring specific mRNA in a microarray analysis) and by measuring immunoglobulins concentration.

2.2 Study Endpoints

2.2.1 Primary Endpoint

The primary endpoint is initial parasitological cure at day 29 and is defined as absence of parasites in tissue aspirate at day 29.

2.2.2 Secondary Endpoint(s)

Relapse-free survival at 12 months as defined as

The patient being alive and disease free from day 29 (if initially cured) or day 58 (if they received an extended treatment) and remains disease free until the last follow-up assessment (i.e. day 390).

Safety

Assessment of safety during treatment and follow-up based on clinical adverse events, laboratory parameters during treatment and 1 month (after end of initial treatment or after end of extended treatment) follow-up.

3. Study design and study design rationale

.

3.1. Study design

This will be a randomized, parallel arm, open-label clinical trial to assess the safety and efficacy of the combination of Ambisome® plus miltefosine and Ambisome® alone for the treatment of VL in HIV co-infected patients in Ethiopia.

3.2. Study duration and duration of subject participation

There will be two recruitment sites in Ethiopia (i.e. Gondar, Abdurafi). Additional sites may be considered in the event that patient recruitment takes longer than expected.

The overall study duration is expected to be 30 months, from start of recruitment to final assessment (12 months follow-up) of last patient and at least 18 months is estimated for the recruitment period.

All patients will be hospitalized for approximately 30 days (including baseline assessments and initial treatment period). However, if the tissue aspirate performed on day 29 is positive and the patient is clinically well (i.e. no fever and clinical improvement of VL signs and symptoms compared to baseline), a complete course of the same treatment will be given and the hospitalization will be prolonged until day 58.

3.3. Rationale of study design

Although WHO recommends Ambisome® 40 mg/kg for HIV VL co-infected patients, this is based on limited information from Mediterranean countries; there are no data available from Ethiopia and there is little information available on the efficay of the test treatments and no established standard treatment with a proven efficacy (of at least 90%) in East Africa. It is therefore not possible to design a comparative study as there is insufficient data to set margins for superiority or non-inferiority. The design of the study will therefore focus on establishing efficacy of two potential test treatments for VL in HIV co-infected individuals.

Recent practice by MSF in their treatment centre at Abdurafi using Ambisome® 30 mg/kg demonstrated a cure rate of only 74% in primary VL cases and of 38% in relapse cases. Increasing the dose to 40 mg/kg seems reasonable to achieve better cure rates. There are no other realistic options for monotherapy with the drugs that are currently available. SSG is too toxic and miltefosine while better tolerated is less effective than SSG as monotherapy.

Combination regimens are attractive as they offer potential added effects; preliminary experience from MSF using Ambisome® 30 mg/kg with miltefosine for 28 days increases the initial cure rate in relapse cases to 87%, while in primary VL this remains at 74%[34]. Moreover, the combination seems to be well tolerated. This study aims to identify a treatment regimen is safe and effective for all HIV VL co-infected patients in both primary and relapse cases. While including primary and relapse cases makes the study population more heterogeneous, the outcome of the trial could be applicable to all cases that present. In the final analysis, cure rates for both arms will be stratified in both arms to see differences between primary cases and

relapses. The study will not be powered to detect statistically different differences as for this the sample size would have to be unrealistically much larger.

Relapses are a major problem in HIV co-infected VL patients and a positive TOC is an important risk factor. For this reason, the study will provide extended treatment with the same regimen (with same dosage and duration) for patients with a positive parasitology examination at day 29 but feeling well (i.e. no fever and clinical improvement of VL signs and symptoms compared to baseline). This may provide additional information on the total quantity of drug that is needed to achieve a negative parasitology examination and thus minimize the risk of relapse.

The study is designed and will be analysed according to group-sequential methods, specifically the triangular test. For ethical reasons, it has become common practice in clinical trials to perform interim analyses or use multistage designs to ensure that either harmful or ineffective treatments are discontinued as early as possible. These planned interim analyses however suffer from low power. Sequential analyses are designed to allow for repeated testing throughout the trial recruitment period while maintaining good statistical properties (pre-specified type I and type II error) and reducing the necessary sample size. Sequential trials, where the data is analysed after every patient is recruited, can be difficult to achieve in many settings. Group sequential trials were developed to allow for discrete data analysis after a pre-specified number of patients are recruited. The triangular test is one way of analysing group sequential trials and uses straight line stopping boundaries.

Follow-up: all patients will be followed until 12 months after the end of initial treatment; although the follow-up time for extended or rescue treatment may be less than 12 months (i.e. all followed up by day 390).

All patients that achieves a negative parasitology examination (including patients receiving rescue medication) will be offered to start secondary prophylaxis with monthly pentamidine 4 mg/kg IM if they have a CD4 count <200 cells/mL and no contraindications to this drug. The secondary prophylaxis will be stopped if CD4 count is over 200 cells/mL at day 390 assessment. It could also be stopped in case of relapse, adverse events requiring discontinuation of medication or if the patient requests to stop the prophylaxis.

Pentamidine injections will be given for a maximum duration of 18 months.

Figure 1- Overall study design

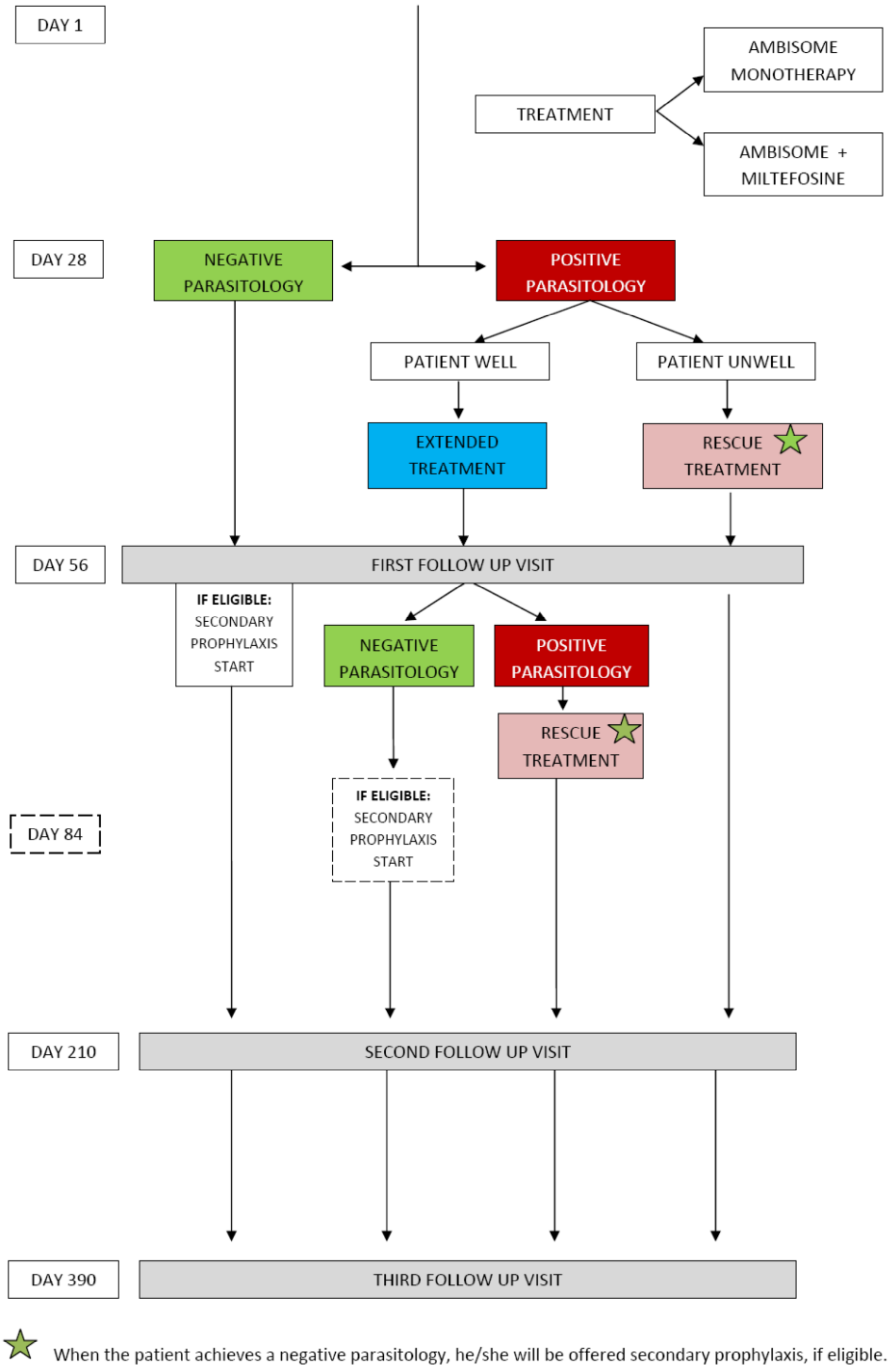

4. Selection of Subjects

The following eligibility criteria were designed to select subjects for whom the protocol treatment is considered appropriate. All relevant medical and non-medical conditions should be taken into consideration when deciding whether this protocol is suitable for a particular subject. Eligibility criteria may not be waived by the investigator. Any questions regarding a subject's eligibility should be discussed with DNDi Medical Coordinator prior to subject's enrollment.

4.1. Inclusion criteria

Subjects must meet **all** of the following inclusion criteria to be eligible for enrollment into the study:

- Confirmed HIV positive test (2 rapid diagnostics tests (RDTs) performed in parallel followed by a confirmatory ELISA test).
- Diagnosis of VL (first episode or relapse) confirmed by bone marrow or spleen aspirate.*
- Male and female age: 18-60 years.
- Written informed consent from the patient.**

4.2. Exclusion criteria

The presence of any of the following will exclude a subject from study enrolment:

- Women of child-bearing potential (defined as women who have achieved menarche) who are not using an assured method of contraception or are unwilling to use an assured method of contraception for the duration of treatment and four months after.***
- Pregnant women or breast-feeding mothers.
- Patients with grade 2 or 3 post kala-azar dermal leishmaniasis (PKDL) lesions
- Clinical symptoms of chronic underlying disease such as severe cardiac, renal or hepatic impairment.
- Known hypersensitivity to AmBisome® and/or miltefosine.
- Patients receiving allopurinol treatment

** Patients with a platelet count less than 40,000/ µL or Hemoglobin (Hb) less than 5g/dL or not palpable spleen or bleeding tendency (e.g. epistaxis) should be diagnosed by bone marrow rather than spleen aspirate. Ideally, the same type of tissue aspirate should be performed at screening and at day 29 assessment (except if not clinically indicated). During follow up, a spleen aspirate can be performed, if required, provided there is recovery of the platelet count, Hemoglobin and/or bleeding tendency.*

*** If the patient is illiterate an impartial witness should be present during the consenting procedure and should also sign.*

**** Women who are sexually active and not using an assured method of contraception i.e. either IUCD, medroxyprogesterone acetate (Depo-Provera) but who wish to participate in the trial must sign an additional consent form and agree to receive a long-acting assured form of contraception. Oral contraceptives are not considered adequate in this context because of the high prevalence of vomiting and diarrhoea associated with miltefosine treatment. As these patients are HIV positive, the above contraceptive methods would be in addition to the use of condoms which should be normal practice and will be encouraged throughout the study.*

5. Schedule of events

Protocol Activities	Screening (day)	Treatment (day)			Day 29	Follow-up Period (days post initial treatment)				
							For patients receiving extended treatment			
	Day – 7 to Day 0	1	3	10	Day 29	Day 58 ± 10d	Day 58	Day 86 ± 10d	Day 210 ± 28d	Day 390 ± 56d
Consent Form	x									
Clinical Assessment [1]	x		x	x	x	x	x	x	x	x
PKDL	x				x	x	x	x	x	x
Full Blood Count	x		x	x	x	x[2]	x	x[2]	x	x
Urea, creatinine	x		x	x	x	x[2]	x	x[2]		
Liver function (Bilirubin, AST, ALT)	x		x	x	x	x[2]	x	x[2]		
CD4 count	x				X[3]	x[2]	x	x[2]	x	x
Viral load	x								x	x
Tissue aspirate [4]	x				x[5]	x[2]	x	x[2]	x[2]	x[2]
Start of VL treatment		x								
Treatment period - Adverse Events monitoring [6]		←<br>←- -				→	- -	- - →		
Concomitant Medications	←							→		
**Secondary Prophylaxis**										
Start						x[7]		x[8]		
Glucose[6, 9]						←		←- -		→
Urea, creatinine + Liver function (Bilirubin, AST, ALT)[10]									←	→
Safety monitoring (SAEs only) [5]						←		←- -		→

1 Includes weight, height, (screening only) vital signs, VL clinical signs and symptoms.
2 Performed only when clinically indicated.
3 CD4 count not performed in case of extended treatment is needed.
4 Tissue aspirate: This will be a spleen aspirate; if the spleen is not palpable a bone marrow aspirate will be done
5 Ideally, the same type of tissue aspirate should be performed at screening and at day 29 assessment (except if not clinically indicated).
6 Dashed line arrow refers specifically to patients receiving an extended treatment. Solid-line arrow refers to patients cured at day 29 assessment.
7 For patients that are parasite negative at day 29 with CD4 count < 200 cells/mL and with no known contra-indication to Pentamidine. Patients need also to be available for the monthly follow-up visits to receive the secondary prophylaxis.
8 For patients that are parasite negative at day 58 after an extended treatment with CD4 count < 200 cells/mL and with no known contra-indication to Pentamidine. Patients need also to be available for the monthly follow-up visits to receive the secondary prophylaxis.
9 At each monthly visit, glucose level will be monitored.
10 Clinical chemistry tests will be performed at 6 months and 12 months after the start of the secondary prophylaxis.

Other analyses performed at Gondar site only on a sub-group of patients *								
**Protocol activities**	**Screening**	**Treatment (day)**			**Day**	**Follow-up Period (days post initial treatment)**		
		**1**	**3**	**10**	**29**	**58**	**210**	**390**
Cytokines, immunoglobulins	x			x	x		x	x
Microarray	x			x	x		x	x
Electrolytes (K+)	x			x	x			
PK samples MF (DBS)	*Please refer to the detailed schedule section 8.6, page 40 & 41*							
PK samples L-Amb (DBS/plasma)	*Please refer to the detailed schedule section 8.6, page 40 & 41*							
PK samples: ARV: peak-trough (DBS)	*Please refer to the detailed schedule section 8.6, page 40 & 41*							

***** Sub-group of patients defined as followed:
- Cytokines, Immunoglobulines: 30 patients/arm (15 patients/arm as a minimum).
- Microarray: 30 patients/arm (15 patients/arm as a minimum).
- Pharmacokinetics (PK): 30 patients/arm (15 patients/arm as a minimum).

6. Enrolment procedures

A computer generated, randomization code will be used for patient treatment allocation to one of the two treatment arms. The randomization will be stratified according to the centre (i.e. Abdurafi and Gondar) as well as by whether or not VL occurred previously (i.e. primary and relapse case).
Two sets of individual, opaque, sealed and sequentially numbered envelopes will be provided to each trial site, one envelope per patient. One set will be used for primary VL cases and the second one for relapse patients.
Eligible patients who fulfill all the inclusion criteria and have none of the exclusion criteria, and from whom informed consent has been obtained, will be randomized to one of the treatment regimens using the sealed envelopes.

The investigator should open the lowest numbered envelope from the correct set (i.e. primary or relapse). The patient receives the treatment written on the card inside. The card must then be replaced in the envelope and retained for drug accountability and monitoring purposes. This method of treatment concealment will minimise selection bias as the investigator will not know the allocation of treatment for a specific patient until the envelope is opened.

7. Treatments

7.1. Investigational Product

<u>Name</u>: **Liposomal Amphotericin B (AmBisome®)**
<u>Class</u>: a macrocyclic, polyene antifungal antibiotic produced by *Streptomyces nodosus*.
<u>Mechanism of action</u>: affects sterol biosynthesis, disrupting the parasite membrane.
Commercial source: Gilead.
<u>Product appearance</u>: AmBisome® comes as a sterile lyophilised powder in a 15ml sterile Type 1 clear glass vial containing a yellow powder with the active ingredient amphotericin B 50mg encapsulated in liposomes. The closure consists of a butyl rubber stopper and aluminium ring seal with a removable plastic cap. Vials are packed in cartons of 10, with 10 filters provided.
<u>Administration</u>:

- Add 12ml of water for injection to each AmBisome® vial, to yield a preparation containing 4mg/ml **(do not use saline or any bacteriostatic agents)**. AmBisome® is incompatible with saline and should not be mixed with other drug or electrolyte solution during administration.
- Immediately after the addition of water, shake the vials vigorously for 30 seconds to completely disperse the AmBisome®. Visually inspect the vials for particulate matter and continue shaking until complete dispersion is obtained.
- The infusion providing from 2.00 to 0.20mg of amphotericin per ml is obtained by dilution with 1 to 19 parts respectively, by volume of 5% dextrose injection.
- Withdraw the calculated volume of reconstituted AmBisome® into a sterile syringe.
- Using the 5 micron filter provided, instil the AmBisome® preparation into a sterile container with the correct volume of dextrose injection.

<u>Name</u> : **Miltefosine (Impavido®)**
<u>Class</u>: Phosphocholine analogue.
<u>Mechanism of action</u>: interferes with the synthesis and metabolism of phospholipids. It may also interfere with the parasite's membrane signal transduction, and glycosylphosphatidylinositol anchor biosynthesis.
<u>Commercial source</u>: Paladin labs.
<u>Product appearance</u>: comes as 10mg and 50mg capsules as a pack of 28 or 56 capsules sealed in 4 or 8 aluminium blister stripes, each containing 7 capsules.
<u>Administration</u>: oral, at a dose of 2.5mg/ kg daily for 28 days. For miltefosine, there are no known interactions with other commonly used medications, though this cannot be excluded.

7.2. Doses and treatment regimens

Dosing schedule:

AmBisome® monotherapy:

- Adapted from WHO recommendations for co-infected patients:
  40 mg/kg total dose: IV infusion of 5 mg/kg on day 1-5, 10, 17, 24.

AmBisome® in combination with Miltefosine:

- AmBisome®: 30 mg/kg total dose: IV infusion 5 mg/kg on day 1, 3, 5, 7, 9, 11.

- Miltefosine: every day during 28 days.
  The dose is calculated according to the patient weight:
  - ≤ 25 Kg, the patient receives 50mg (i.e. 1 x 50mg capsule) per day.
  - > 25Kg, the patient receives 100mg (i.e. 2 x 50mg capsules) per day.

Miltefosine capsules should be taken with meals.

Dosages of 2 capsules per day should be divided into 2 individual doses to be taken in the morning and evening. In the event the patient vomits within 1 hour of a dose, that dose should be repeated. In the event of repeated vomiting the patient should be withdrawn from that treatment allocation and treated with rescue medication.

7.3. Drugs labelling, packaging

Commercially available products will be used as all drugs have been registered for VL. Trial specific labeling will be applied prior to use.

7.4. Accountability

All study medications must be kept in a locked room that can be accessed only by the appropriate study personnel. The study medications must not be used for other purposes other than this protocol. Under no circumstances the investigator or site staff may supply study medications to other investigators or sites, or allow the medications to be used other than as directed by this protocol without prior authorization from DNDi. Adequate records on receipt, use, return, loss, or other disposition of medication must be maintained.

7.5. Storage

AmBisome®: The product should be stored at 25°C or below, not frozen or exposed to light. The shelf life is 3 years. Once reconstituted, chemical and physical in-use stability has been demonstrated for 24 hours at 25+/-2°C in vials exposed to ambient light. This is increased to 7 days if stored at 2-8°C. However to avoid contamination, once reconstituted, the product should be stored at 2-8°C and be used within 24hours.

Miltefosine: The product should be stored in the original package and protected from moisture. The shelf life is 4 years. Packaging should be undamaged prior to use.

7.6. Blinding and procedures for unblinding

This is an un-blinded study.

7.7. Concomitant medications

Conditions such as malaria and respiratory tract infections should be treated prior to VL treatment if identified early enough. Exclusion of such common conditions should be undertaken for all VL patients prior to commencement of treatment. However it is expected that some patients may require management of such common conditions during study treatment.

Patients will be started on antiretroviral medication according to the Ethiopian National Guidelines (standard regimen: tenofovir, lamivudine and efavirenz (TDF-3TC-EFV) as soon as their condition permits and the ART adherence sessions have been completed; i.e. normally after 2-4 weeks. Those who are already on ART will continue the same regimen throughout the study, unless there is a clinical indication to change. Some patients will use other ART regimens, with zidovudine instead of tenofovir or nevirapine instead of efavirenz. These regimens will not be changed unless there is a clinical indication to do so.

Cotrimoxaxole preventive therapy (CPT) is indicated for all HIV infected patients according to the Ethiopian National Guidelines; those who are not yet on CPT will be given cotrimoxazole 480 mg bd as soon as they are able to take oral medication.

7.8. Secondary prophylaxis

Secondary prophylaxis of monthly pentamidine (4mg/kg IM) will be offered to all patients that clear parasites independently of when this is achieved (i.e. initially cured on day 29, extended treatment cured on day 58 or relapse patient that achieved cured at any point in time). Patients will be eligible for the secondary prophylaxis if their CD4 count is < 200 cells/mL and there is no contraindication (e.g. renal failure, diabetes mellitus, known hypersensitivity) for the drug used. Secondary prophylaxis will normally be continued till the patient has sufficient CD4 recovery.(i.e. over 200 cells/mL at day 390 assessment). Prophylactic treatment can be continued after the study end (day 390) if required. However, it would be stopped in case of relapse, adverse events requiring discontinuation of medication or if the patient requests to stop the prophylaxis.

The prophylaxis will be started one month after the end of the treatment and will be dispensed every month up to a maximum of 18 months.
At each of the monthly visit the glucose level will be monitored. Moreover, liver and kidney function will be performed at day 210 and 390 assessments.

7.9. Rescue medication

Patients will be given rescue medication for the following reasons:

- positive test of cure at day 29 and patient unwell
- failure to respond to the extended treatment at day 58
- relapse during follow-up period

The attending physician has discretion to give rescue medication.

Any patients who receive rescue medication at any point will be considered a treatment failure.

8. Study Assessments

8.1. Timing of Assessments

Baseline assessments will be carried out within 7 days before VL treatment starts.

Assessments will be timed at day 1, 3, 10, 29, 58, 86 (for extended treatment only), 210 and 390 and will include clinical, parasitological, haematological, biochemical assessments.
A table with scheduled events is listed under section 5.

Assessments on day 58 (86 for extended treatment only), 210 and 390 post treatment will require some flexibility on dates due to patient travel, visit windows for each will be as follows- for day 58/86 (+/- 10 days); day 210 (+/- 1 month) and day 390 (+/- 2 months).

8.2. Baseline Assessments

Baseline assessments will include anthropometric, clinical and laboratory evaluations.

8.3. Assessment of Efficacy

Clinical assessment
The clinical evaluation will involve measuring temperature (axiliary), spleen size, liver size, body weight at screening, on day 3, 10, 29 and at all follow-up assessments (i.e. 58, 86, 210 and 390 days).
Height will be measured at baseline only.
The size of the liver will be measured in the midclavicular line for its total span; the spleen size will be measured from the left costal margin on the anterior axillary line to the tip of the spleen medially.

Post-kala-azar dermal leishmaniasis (PKDL)

Patients will be monitored closely for post-kala-azar dermal leishmaniasis (PKDL) through the course of the study. Diagnosis will be made clinically based on the typical appearance and distribution of the rash. Presence of PKDL will be noted at screening, day 29 and during follow-up assessments on day 58, 210 and 390. Grading will be noted during the assessment times as follows:

- mild (Grade 1)
- moderate (Grade 2)
- severe (Grade 3).

A patient with grade1 PKDL has lesions mainly on the face and head, with others scattered on arms, chest and back. In grade 2, the upper part of the chest and arms are also affected. In grade 3, lesions may be found on the whole body.
Patients who develop severe grade 2 or grade 3 or have mucosal and/or eye involvement (any grade) will require treatment with rescue medication at the discretion of the attending physician.

When PKDL occurs while the patient is still receiving study medication, the study medication will be continued. If at day 29 assessment, a patient has severe grade 2 or grade 3 PKDL or PKDL with mucosal and/or eye involvement (any grade), these patients will receive rescue treatment and will be considered treatment failures.

Laboratory assessment
Parasitological assessment will be done at baseline and at day 29; it will also be done at day 58 for patients who have an extended treatment. During the follow-up, it will only be performed when clinically indicated. It will be a spleen aspirate or a bone marrow aspirate if the spleen is not enlarged. Aspirates are smeared on slides, stained and graded according to the standard logarithmic criteria as defined in the below table:

**Count Oil Immersion x 100**	
**6+**	> 100 parasites per field
**5+**	10-100 parasites per field
**4+**	1-10 parasites per field
**3+**	1-10 parasites per 10 fields
**2+**	1-10 parasites per 100 fields
**1+**	1-10 parasites per 1000 fields
**0**	0 parasite per field

8.4. Assessment of Safety

Clinical Assessment

The clinical evaluation will involve measuring pulse rate, blood pressure at screening, on day 3, 10, 29 and at all follow-up assessments.

Laboratory examinations

Blood will be analyzed for haemoglobin, WBC, RBC, platelets at screening, on day 3, 10, 29, 58 (for patients with negative parasitology at day 29 - done only if clinically indicated), 86 (extended treatment – done only if clinically indicated), 210 and 390. Urea, creatinine, and liver function (AST, ALT, bilirubin) tests will be performed at screening, on day 3, 10, 29, 58 (for patients with negative parasitology at day 29: done only if clinically indicated). Urea, creatinine and liver function will also be mandatorily be performed at day 210 and 390 assessments, if the patient receives the secondary prophylaxis.

Response to ART

CD4 count will be measured at baseline, on day 29 and on day 58 (86), 210 and 390. The analysis will be performed at the local lab of each of the two sites.

The HIV viral load will be measured during screening, on day 210 and 390. A 0.5 mL plasma sample will be collected and stored at -20°C and shipped to the Amhara region Regional Laboratory, at Bahir Dar for analysis.

8.5. Follow up assessments

All patients will undergo 3 follow-up assessments at day 58, (day 86 - extended treatment only), 210 and 390 post initial treatment independently of their outcome (i.e. patients initially cured at day 29, patients receiving extended treatment and/or rescue treatment).

8.6. Other assessments

Measurement of immunoglobulins and cytokines

Immunoglobulins and cytokines will be measured in plasma by ELISA. A 3 ml EDTA blood sample will be collected at screening and on day 10, 29, 210, 390 for measurement of immunoglobulins (IgG [total and IgG 1-4 subtypes], IgM, IgA, IgD and IgE levels) and cytokines (IL-2, IL-4, and IL-10 and gamma-interferon).
The samples will be collected at Gondar site only. A minimum of 15 patients/arm will be included up to 30 patients/arm.
A 6.5 mL blood sample will be stored at the trial sites at -20°C and shipped and analyzed as a batch at the Department of Immunoparasitology, Addis Ababa University.

Microarray

As the study of any multifactorial phenomenon, and of a complex co-infection such as HIV-VL in particular, may be too complex a challenge to address using conventional single-or-limited parameter experimental setups, we aim to perform genome-wide transcriptome/microarray analysis, which maps the expression of all genes in the human genome in a single experiment. This will allow us to reliably identify and validate biomarkers for specific modes of disease progression and/or

therapy response and to generate novel hypotheses on the pathogenesis of HIV/VL co-infection. Based on published data and ITM experience, we will particularly focus on:

a) Factors associated with the Th1-Th2 balance. Cytokine and particularly chemokine (which are almost exclusively regulated at the level of gene expression) networks will be analysed for polarisation towards a Th1 or Th2 environment, and possible causative factors for this polarisation will be mapped.
b) Factors which are indicative for bacterial translocation from the gut to the periphery and consequent immune activation (e.g. the sCD14-TLR4-TNF axis), which may result from both HIV- and/or VL-induced damage to the gut mucosa. Such immune activation would go a long way towards explaining the observed incomplete restoration of anti-VL immune responses: due to chronic overstimulation, lymphocytes remain exhausted and cannot mount an adequate anti-VL response.
c) Factors which are indicative of increased myeloid-derived suppressor cell activity (such as he iNOS, NADPH oxidase and arginase-1 effector molecules and HIF1a, STAT3 and C/EBPb transcription factors), an immune suppressive cell type which is well-documented in cancer biology and which has been shown to be induced in animal models of VL. Induction of a dominant MDSC population could effectively prevent the immune system from coming fully online under ART and could thus preclude complete parasite clearance during anti-VL therapy.

The samples for the microarray analysis will be collected at Gondar site only.
A minimum of 15 patients/arm will be included up to 30 patients/arm.
A 2.5 ml of blood sample will be collected in PAXgene tubes at screening and on day 10, 29, 210, 390. The samples will be stored at -20 ^{0}C and then be shipped and analyzed as a batch at ITM, Antwerp.

Pharmacokinetics:
Assessment of drug-drug interaction between VL treatment given and antiretroviral drugs.

Amphotericin B

Limited clinical studies have been done on the pharmacokinetics of (liposomal) amphotericin B in the treatment of visceral leishmaniasis. The pharmacokinetics of liposomal amphotericin B have been shown to be non-linear, pointing at saturation of kinetics, which makes extrapolation of previous findings done with lower doses of liposomal amphotericin B as antifungal difficult[35]. Interactions between amphotericin B and antiretroviral drugs have not been well studied, also not in the treatment of other HIV-co-morbidities[36]. Amphotericin B has been associated with modulation of metabolic CYP450 enzyme activity, which has been implicated most notably with changes in PI and NNRTI metabolism and thus HAART exposure[37] [38]. Some data suggest that this effect is less significant for the liposomal formulation, although good studies are absent for liposomal amphotericin B. Little is known about the exact pharmacokinetics of high-dose liposomal amphotericin B, although it tends to accumulate in the liver, the metabolic site of action for most PIs and NNRTIs. Also, increased rates of renal toxicity have been observed when amphotericin B is combined with tenofovir and therefore concomitant or even subsequent

administration of these compounds should be closely monitored[36]. The combination of zidovudine and amphotericin B has been associated with additive myelosuppressive effects, particularly anemia[36].

For (liposomal) amphotericin B, a high variability in pharmacokinetics has previously been described between different study populations reflecting the possible influence of a variety of pathophysiological changes on its pharmacokinetics with a tendency of obviously lower levels in critically ill patients[39]. However, nothing is known about the effect of HIV-VL morbidity on the pharmacokinetics of liposomal amphotericin B. When liposomal amphotericin B is freed from the liposomes, 90% of the drug is bound to proteins (albumin). Interactions on this level, certainly in malnourished patients with lower blood protein levels, can be expected when combined with other highly protein-bound drugs like efavirenz (99%), tenofovir (90%) or miltefosine (95-98%). Clinical as well as intrinsic metabolic effects of a change in bound/unbound drug fraction are unknown as well as the effect on tissue/intracellular distribution. Amphotericin B is not known to be a substrate, inducer or inhibitor of any of human multi-drug resistance transporters and no interactions are expected on this level with HAART medication or miltefosine.

Miltefosine

In general, pharmacokinetics of miltefosine in HIV-positive patients are unknown and nothing is clinically known about drug-drug interactions between HAART and miltefosine. Miltefosine is metabolized, mainly, by phospholipase D and is not known to be a substrate for any of the cytochrome P450 enzymes (CYP450)[40]. Drug-drug interactions on the CYP450 level are thus not expected. Nevertheless, miltefosine can cause (severe) hepatotoxicity in a small selection of patients and might theoretically influence drugs which are mainly hepatically metabolized, like almost all PIs and NNRTIs. Miltefosine has been suggested *in vitro* as a substrate for the multi-drug-resistance transporter P-glycoprotein (P-gp)[41], which is modulated intensively by e.g. ritonavir. Also lamuvidine and efavirenz are known to be important substrates for P-gp and are both (relatively low) inducers of P-gp (~2-fold increase of activity *in* vitro)[42]. An increased activity of P-gp could lead to a reduced absorption of drug substrates from the gastrointestinal tract (lower plasma levels), but also decreased intracellular levels of drug substrates.

Similar to amphotericin B, interactions with HAART and amphotericin B on the level of protein-binding can be expected as well since miltefosine is 95-98% protein-bound. A small relative change in the fraction unbound to protein, would increase free levels of miltefosine dramatically and might have a high negative impact on the accumulation of miltefosine.

For miltefosine and antiretroviral drug levels, dried blood spots will be collected using Whatman 903 protein saver card. For Ambisome® levels, a 2 ml EDTA blood sample will be collected. These samples will be stored at -20 ^{0}C and shipped to the Slotervaart Hospital, Amsterdam where they will be analyzed by validated LC/MS/MS methodology.

The PK samples will be collected at Gondar site only. A minimum of 15 patients/arm will be included up to 30 patients/arm.

The specific PK timepoints are detailed in the two tables below according to each of the treatment arms:

Schedule of pharmacokinetic events

Arm 1: Monotherapy Ambisome® (40 mg/kg total dose)

Activities	Matrix	Volume per sample	Screening (day)	Treatment (day)								Follow-up Period (months post treatment)		
			Day – 7 to Day 0	1	2	3	4	5	10	17	24	Day 58	Day 210	Day 390
**Rx - Ambisome® infusion**				X	X	X	X	X	X	X	X			
**PK AmphoB**														
**PK AmphoB[1]**	*EDTA plasma*	2 mL blood		X X X[1]							X X X[1]			
**Total volume blood taken for PK in mL**				10							10			
**PK EFV/NVP/LPV/RTV**														
**PK EFV/NVP/LPV/RTV[2]**	*DBS (duplicate)*	~50 µL blood		X X[2,3]							XX[2]	XX[2]	XX[2]	XX[2]
**Total number of DBS (incl duplicates)**				4							4	4	4	4

1 For a full curve: 2, 6 and 24h post-infusion (3 timepoints), 2mL of whole blood per sample for each timepoint → 500 µL plasma yields two duplicate samples of 250 µL → 50 µL will be used for total amphotericin B determination, 200 µL will be used for free amphotericin B determination (ultracentrifugation).

2 Peak (toxicity) and trough (efficacy) levels for ARV drugs: steady-state concentration before taking dosage, peak levels for EFV/NVP/LPV/RTV approx. 4-5h after dose intake. EFV/NVP/LPV/RTV can be measured simultaneously in the same sample, since they have similar T_{max}'s.

3 For PK of ARV's DBS's will be taken on the day of treatment initiation:
a) if patient is already on ARV's before study inclusion this will indeed be day 1
b) if ARV's will be initiated during study, DBS's for ARV PK will be sampled on the first day of ARV treatment, e.g. if ARV's are initiated on day 10, sampling will also be done on day 10.

In case of extended treatment, an additional sampling point will be taken **<u>on the last AmBisome infusion, prior to dosing</u>** for AmphoB & EFV/NVP/LPV/RTV.

Arm 2: Combination therapy Ambisome® (30 mg/kg total dose) + 28 days of miltefosine

Activities	Matrix	Volume per sample	Screening (day)	Treatment (day)						End of Treatment (day)	Follow-up Period (days post treatment)		
			Day – 7 to Day 0	1	3	5	7	9	11	29	Days 58	Days 210	Days 390
**Rx - Ambisome® infusion**				X	X	X	X	X	X				
**Rx – Miltefosine**				Start →						End			
**PK AmphoB**													
**PK AmphoB**[1]	EDTA plasma	2 mL blood		X X X[1]					X X X [1]				
**Total volume blood taken for PK in mL**				10					10				
**PK EFV/NVP/LPV/RTV / PK Miltefosine**													
**PK EFV/NVP/LPV/RTV**[2]	DBS (duplicate)	~50 µL blood		X X[2,3]						X X[2]	X X[2]	X X[2]	X X[2]
**PK Miltefosine**	DBS (duplicate)	~50 µL blood	X						X	X[4]	X	X	
**Total number of DBS for PK (incl duplicates)**			2	4					2	6	6	6	4

1 For a full curve: 2, 6 and 24h post-infusion (3 timepoints), minimally 1.0 mL (preferably 2 mL in case of spilling etc.) of whole blood per sample for each timepoint → 500 µL plasma yields two duplicate samples of 250 µL → 50 µL will be used for total amphotericin B determination, 200 µL will be used for free amphotericin B determination (ultracentrifugation).

2 Peak (toxicity) and trough (efficacy) levels for ARV drugs: steady-state concentration before taking dosage, peak levels for EFV/NVP/LPV/RTV approx. 4-5h after dose intake. EFV/NVP/LPV/RTV can be measured simultaneously in the same sample, since they have similar T_{max}'s.

3 For PK of ARV's DBS's will be taken on the day of treatment initiation:
a) if patient is already on ARV's before study inclusion this will indeed be day 1
b) if ARV's will be initiated during study, DBS's for ARV PK will be sampled on the first day of ARV treatment, e.g. if ARV's are initiated on day 10, sampling will also be done on day 10.

4 Blood drawn at day 29 for full blood count will also be used (no additional samples) to correlate Miltefosine level in DBS and plasma.

In case of extended treatment, an additional sampling point will be done **on the last AmBisome infusion, prior to dosing** for miltefosine, AmphoB and EFV/NVP/LPV/RTV.

8.7. Adverse event definitions and reporting

8.7.1. Adverse Event definition

An adverse event will be defined as any untoward medical occurrence (any unfavourable and unintended sign, symptom or disease, including an abnormal laboratory finding) in temporal association with the use of the investigational treatment and may or may not be causally related to it.
Abnormal laboratory results will be reported as adverse events if the abnormality occurs or worsens after institution of the study treatment, and if they require clinical intervention or further investigation, unless they are associated with an already reported clinical event.

All safety events that require reporting to the Ethiopian authority (i.e. NHRERC and FMHACA) will be made according to the national regulations.

8.7.2. Serious Adverse Event

An adverse event will be defined as serious if it is

- fatal
- life-threatening
- requires or prolongs hospitalization
- results in persistent or significant disability
- is a congenital anomaly/birth defect
- results in an important medical event that may not be immediately life threatening or does not directly result in death or hospitalization, but which may jeopardize the patient or may require intervention to prevent the other outcomes listed above.

Serious events also include any other event that is defined as serious for the specific purposes of the protocol or which is defined as serious by the regulatory agency in the country in which the event occurred.

Any prolongation of the hospitalization that is not related to a medical event but related to any social and/or financial reasons will not be considered as an SAE.

8.7.3. Eliciting Adverse Event information

The investigator is required to report all directly observed adverse events and all adverse events spontaneously reported by the trial subject using concise medical terminology. In addition, each trial subject will be questioned about the occurrence of adverse events on a daily basis during the hospitalization period (day 0-29; or until day 58 in case of extended treatment), and 1 month after the stop of treatment (i.e. day 58 or day 86 in case of extended treatment) with a generic question such as "have you felt different in any way/ had any problems since starting the new treatment/the last assessment?" If the response is "Yes", the nature of the event, the date and time (where appropriate) of onset, the duration, maximum intensity (see below) and relationship to treatment will be established (see below). Details of any

dosage/schedule modification or any corrective treatment will be recorded on the appropriate pages of the CRF.

8.7.4. Adverse Event reporting period

The adverse events reporting period for this trial begins:

- Upon administration of the first dose of study medication for non-serious and serious events and ends 1 month after the stop of the treatment when a further safety evaluation occurs.

All adverse events that occur during the adverse event reporting period specified in the protocol must be reported to DNDi, whether or not the event is considered related to study medication.

In case a patient receives the secondary prophylaxis, there will be another period of safety reporting. The reporting period starts upon administration of the first dose of Pentamidine up to a week after the last injection of Pentamidine that occurs during the study period. Only Serious Adverse Events will be collected during this period.

8.7.5. Adverse Event reporting requirements

Information on adverse events must be evaluated by a physician. Each adverse event is to be classified by the investigator as serious or non-serious. This classification will determine the reporting procedure for the event.
All serious adverse events (SAE) are to be reported immediately (within 24 hours of awareness of SAE by the investigator) either by mail to the following address; SAE_VL_Africa@dndi.org or by phone to the DNDi medical coordinator, using the SAE report form. This includes a description of the event, onset date and type, duration, severity, relationship to study drug, outcome, measures taken and all other relevant clinical and laboratory data. The initial report is to be followed by submission of additional information (follow-up SAE form) as it becomes available. Any follow-up reports should be submitted as soon as possible, and if possible within 7 working days.
Serious adverse events should also be reported on the clinical trial adverse event case report form (CRF). It should be noted that the form for reporting of SAE (SAE form) is not the same as the adverse event section of the CRF. Where the same data are collected, the two forms must be completed in a consistent manner, and the same medical terminology should be used.
Non-serious adverse events are to be reported on the CRF, which is to be submitted to DNDi as specified in the Study Documentation section of this protocol. In the CRF, a given adverse event will be recorded only one time per patient, and the severity recorded will be the maximum level reached. If several distinct episodes of the same condition occur, their number will be recorded in the CRF.

8.7.6. Grading of Adverse Event severity

Clinical adverse events and laboratory parameters will be graded by investigator using the Common Toxicity Criteria for Adverse Events (CTC AE) version 4 (May 2009) so that comparisons can be made between treatment arms. This specific

adverse event severity grading scale is used in order to standardize reporting between investigators and sites.
In the case that an AE is not listed, the assessment of intensity/severity will be based on the investigator’s clinical judgment. The maximum intensity/severity experienced should be recorded and will be assigned to one of the following categories.

- MILD - Does not interfere with patient's usual functions
- MODERATE - Interferes to some extent with patient's usual functions
- SEVERE - Interferes significantly with patient's usual functions

It is to be noted the distinction between severity and seriousness of adverse events. A severe adverse event is not necessarily a serious event.

8.7.7. Adverse Event causality assessment

For both serious and non-serious adverse events, the investigator is required to assess the possible relationship between the adverse event and the study drug, i.e. to determine whether there exists a reasonable possibility that the study drug caused or contributed to the adverse event. Causality will be listed as not related, unlikely, possible or probable.
To help investigators with the decision binary tree (bullet points below) in the evaluation of causality, the CIOMS VI group recommends that investigators be asked to consider the following before reaching a decision:

- Medical history
- Lack of efficacy/worsening of existing condition
- Study medications
- Other medications (concomitant or previous)
- Withdrawal of study medication, especially following trial discontinuation / end of study medication
- Erroneous treatment with study medication (or concomitant)
- Protocol related procedure

The decision to suspend, and resume treatment or to permanently interrupt treatment due to an adverse event will be left to the clinician in charge. This decision will be taken based on the patients best interests.

8.7.8. Exposure in utero

All women who are pregnant are to be excluded from this study. This is specifically due to the teratogenic potential of miltefosine (demonstrated in rats). The manufacturer recommends that effective contraception should be used during and up to 3 months after treatment of miltefosine but we will extend it to 4 months according to new available data[43]. However, in the unlikely event that any trial subject becomes or is found to be pregnant while receiving an investigational drug or within 90 days of discontinuing the investigational drugs, the investigator must submit the event on an SAE form. This must be done irrespective of whether an adverse event has occurred. The information submitted should include the anticipated date of delivery. The investigator will follow the subject until completion of the pregnancy or until pregnancy termination (i.e., induced / spontaneous abortion). The investigator will provide pregnancy outcome information as a follow up to the initial SAE form. In the case of a live birth, a pediatrician should assess the infant at the time of birth and submit a report.

8.7.9. Adverse event follow up

All adverse events should be followed until they are resolved or the investigator assesses them as chronic or stable or the subject participation in the trial ends (i.e., until a final report is completed for that subject). In addition, all serious adverse events and those non-serious events assessed by the investigator as possibly related to the investigational drug must continue to be followed even after the subject participation in the trial is over. Such events should be followed until they resolve or until the investigator assesses them as “chronic” or “stable.” Resolution of such events is to be documented on the CRF/SAE form as appropriate.

9. Withdrawal criteria

If a subject withdraws from the study, the reason must be noted on the CRF. If a subject is withdrawn from the study because of a treatment limiting adverse event, thorough efforts should be made to clearly document the outcome.

A subject should be withdrawn from the trial treatment if, in the opinion of the investigator, it is medically necessary and in the best interests of the subject, or if it is the wish of the subject. If a subject does not return for a scheduled visit, every effort should be made to contact the subject. In any circumstance, every effort should be made to document subject outcome, if possible.

If the subject withdraws consent, no further evaluations should be performed and no attempts should be made to collect additional data, with the exception of follow-up safety data on events that occurred prior to the patient withdrawing their consent. Such information should be collected if possible.

All patients who are classified as a treatment failure and who receive extended treatment or rescue treatment will be followed for 12 months.

9.1. Rules in case of treatment suspension or interruption

In the event that treatment is interrupted (e.g. due to an adverse event), the decision to resume treatment will be taken by the site Principal Investigator.

If miltefosine treatment is interrupted for more than 3 days and requires additional VL treatment (other than that described in the protocol) they will be considered a treatment failure for the purposes of the ITT analysis and excluded from the per protocol analysis. If interruption was for 3 or less days, treatment will start again till the complete dose is administered.

9.2. Rules for permanently interrupting study treatment

If a subject is withdrawn from the study before the full course of the treatment is completed, the day 29 procedures should be completed (i.e. parasitology assessment) unless contraindications are present. The physician must make all necessary arrangements to ensure that the subject receives the appropriate treatment for the relevant medical condition (e.g. with drug/s currently recommended

by the national policy).

10. Data Analysis and Statistical Methods

10.1. Sample size determination

The sample size will be determined according to the sequential design for the primary outcome to be measured at day 29.

Since we are using a sequential approach, the sample size is not defined or known in advance. The maximum sample size needed per arm would be 66 patients (132 total patients) and the maximum analyses performed would be 7 (see Graph 1, upper horizontal axis). The actual sample size may be less than this, with its value depending on the value of the proportion cured.

Graph 1: Triangular region for study arms showing the boundaries for analysing the sequential trial using the Triangular Test, with the following parameters (p_0 =0.75, p_a=0.9, α=0.05, β =0.05 and n=10).

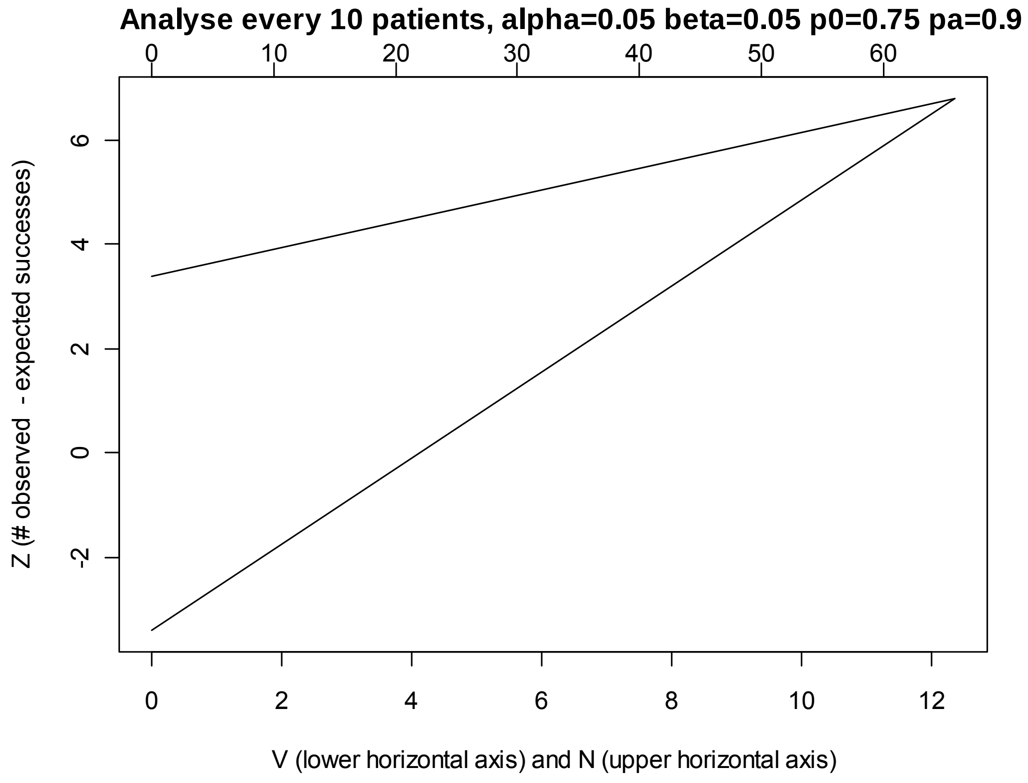

10.2. Definition of study populations included in the analysis

For the sequential and primary analysis (day 29), an intention to treat (ITT) and per protocol (PP) analysis will be performed.

To take account of missing data, each of the above analysis sets (ITT and PP), the efficacy endpoint will be calculated in two ways:

- complete case analysis: excluding patients with missing efficacy data from estimation of efficacy
- worst case analysis: patients with missing efficacy data will be allocated efficacy results based on their 'worst-case' scenario i.e. treatment failure.

Missing outcome data on the efficacy endpoints could arise if a patient withdraws consent, is lost to follow-up, or has an exam omitted by investigator oversight.

The ITT complete case analysis will be primary.

10.3. Subject Disposition

The number of patients who were screened, failed screening, randomised, completed the treatment period, completed the follow-up, early terminations and reasons for early termination of study will be summarized by number of patients (n) and percentages (%).

10.4. Baseline

The continuous variables of the baseline and demographic characteristics will be summarized using number of patients (n), mean, SD, median, minimum and maximum. The categorical variable gender will be summarized using number of patients (n) and percentage.(%).

10.5. Treatment Compliance

All VL drugs will be directly administered (IV) or be directly observed (oral) by study medical staff. All failures with compliance (e.g. vomiting) will be appropriately documented.

10.6. Efficacy Analysis

The primary and secondary endpoints have been described in section 2.2.

The primary endpoint (day 29) analysis will be defined as follows:

- Treatment success: absence of parasites on day 29; hence no rescue medication provided up to this time-point.

- Treatment failure: presence of parasites at day 29 *or* requirement of rescue medication due to lack of clinical improvement/ response *or* withdrawn from the study (e.g. due to a treatment limiting adverse event/ patient absconded) *or* death.

The data will be analysed as proportions according to an intention to treat and per protocol analysis for each treatment arm.

The secondary endpoint (12 months post treatment) analysis will be defined as follows:

- Treatment success: treatment success at day 29 or at day 58 in case of extended treatment and patient being alive and disease free (absence of signs and symptoms of VL or if symptomatic, a negative parasitological assessment by tissue aspirate) at day 390.

- Treatment failure: presence of signs of symptoms of VL, confirmed by presence of parasites in a parasitological investigation. It is mandatory that all patients who have possible presence of the disease have a parasitological assessment (a spleen aspirate or a bone marrow aspirate if the spleen is not palpable).

The primary endpoint will be analyzed following Whitehead (1983) including point and interval estimation of the proportion cured in each treatment arm.

Additional analyses will be conducted to evaluate potential differences in response to treatment between relapse versus primary VL cases, and patients that have previous exposure to ART or not. This will be done at the final sequential analysis (when we reach 66 per arm) and after one year follow up. A detailed description will be included in the statistical analysis plan.

10.7. Safety Analysis

Adverse events will be described on treatment and during follow-up. AEs will be coded using MedDRA and tabulated by severity / CTC V4 AE grading. Causality of AEs will also be tabulated. Serious Adverse Events will be described by individual narratives based on the SAE reports provided by the site investigators. Summary statistics will be tabulated for hematological and biochemical parameters as well as for efficacy of ART (CD4 count, viral load) at each time point.

10.8. Analysis of other endpoints

Other parameters including pharmacokinetics, microarray and cytokine, immunoglobulin levels will be analyzed and reported independently. A summary of results will be included as an appendix to the final clinical study report.

10.9. Interim analysis

There will be sequential analysis every 20 patients (10 patients in each arm), up to completion of the study. A maximum of 7 analysis will be performed. DSMB will consider if both treatment arms should be continued based on pre-defined criteria set in the sequential analysis. DSMB will also review the available safety data (AEs, SAEs) for both treatment arms.

In order for the findings of this study to be generalizable the population included in this study is heterogeneous (i.e. includes relapse and primary VL patients, patients currently on ART and those that will start ART). In order to address potential concerns regarding the heterogeneity of the population and reduce the likelihood of rejecting a treatment that may be effective in a sub section of the population a test to assess heterogeneity will be performed when a treatment is stopped. This test will be

conducted to verify on the available data if there is a signal of interaction ($p < 0.10$ = possible interaction) between strata (primary VL or relapse; patients currently on ART or patients initiated on ART during the study) and treatment (magnitude of the treatment effect is dependent upon strata). In absence of signal of interaction ($p > 0.10$) the magnitude of the difference in success rates between treatments should be relatively homogeneous across strata. In presence of significant signal interaction, the treatment effect will have to be adjusted on strata to know whether both therapeutic strategies have the same success rate. Depending on the outcome of this test for heterogeneity, recruitment may be continued into one stratum. This decision will be taken by the sponsor following consultation with the DSMB and other relevant authorities.

11. Data Safety Monitoring Board

A Data Safety Monitoring Board (DSMB) composed of a minimum of 3 members including a statistician and a physician all of whom must be independent of the investigator and sponsors. DSMB will be set up prior to study initiation. The DSMB will monitor the study in order to ensure that harm is minimised and benefits maximised for the study subjects. They will review the study data after every 20 patients (10 patients in each arm) that have been included (see section: on data analysis) and issue recommendations about the study.

12. Quality Assurance and Quality Control Procedures

12.1. Investigator’s file

The investigator must maintain adequate and accurate records to enable the conduct of the study to be fully documented and the study data to be subsequently verified. These documents include Investigator’s Site File, subject clinical source documents and screening / enrolment logs. The Investigator’s Site File will contain the protocol/protocol amendments, CRF and query forms, IEC and regulatory approval with correspondence, sample informed consent, drug accountability records, staff curriculum vitae and authorization forms and other appropriate document, correspondence etc.

12.2. Case report forms (CRFs)

Data will be collected by laboratory technicians, medical doctors, clinical officers and nurses authorized by the investigator. It will be supervised by the Investigator and signed by the investigator or by an authorised staff member. Study-specific information will be entered into the Case Report Form (CRF). Data that are derived should be consistent with the source documents or the discrepancies should be explained. All CRF data should be anonymised ie identified by study patient number and initials only.

The investigator at each trial site should ensure the accuracy, completeness, legibility, and timelines of all data reported to the sponsor in the CRFs and any other additional information that is required. The investigator is responsible for keeping all

consent forms, screening forms, CRF and the completed subject identification code list in a secure location.

12.3. Source documents

The verification of the CRF data must be by direct inspection of source documents. Source documents include subject hospital/clinic records, physician's and nurse's notes, appointment book, original laboratory reports, ECG, EEG, X-ray, pathology and special assessment reports, signed informed consent forms, consultant letters, and subject screening and enrolment logs.

The investigator must maintain source documents such as laboratory and consultation reports, history and physical examination reports, etc., for possible review and/or audit by DNDi and/or Regulatory Authorities. The Investigator / designee will record the date of each subject's visit together with a summary of their status and progress in the study.

12.4. Record Retention

The investigator must keep all study documents on file for at least 7 years after completion or discontinuation of the study. After that period of time the documents may be destroyed with prior permission from DNDi, subject to local regulations.

Should the investigator wish to assign the study records to another party or move them to another location, DNDi must be notified in advance.

12.5. Monitoring

Monitoring visits to the trial site will be made periodically by DNDi representatives or designated clinical monitors to ensure that GCP and all aspects of the protocol are followed. Source documents will be reviewed for verification of consistency with data on CRFs. The investigator will ensure direct access to source documents by DNDi or designated representatives. It is important that the investigators and their relevant personnel are available during the monitoring visits.

The investigators will permit representatives of DNDi and/or designated clinical monitors to inspect all CRFs, medical records, laboratory work sheets and to assess the status of drug storage, dispensing and retrieval at anytime during the study. The corresponding source documents for each subject will be made available provided that subject confidentiality is maintained in accordance with local regulations and to verify adherence to the protocol and to GCP. It is important that the investigators and other trial site staff are available at these visits.

The monitoring visits provide DNDi with the opportunity to evaluate the progress of the study, verify the accuracy and completeness of CRFs, resolve any inconsistencies in the study records, as well as to ensure that all protocol requirements, applicable regulations, and investigator's obligations are being fulfilled.

It will be the clinical monitor's responsibility to inspect the CRF at regular intervals

throughout the study, to verify the adherence to the protocol and the completeness, consistency and accuracy of the data being entered on them. The investigator agrees to cooperate with the clinical monitor to ensure that any problems detected in the course of these monitoring visits are resolved.

A monitoring visit will take place approximately every 6 weeks at each site. This schedule could be adapted depending on specific recruitment rate at each site.

12.6. Audits and inspections

The trial site may also be subject to quality assurance audits by DNDi or designated representatives and/or to inspection by regulatory authorities or Independent Ethics Committees (IEC).

It is important that the investigators and their relevant personnel are available for possible audits or inspections.

12.7. Data Management

After the CRF has been completed and monitored by the clinical monitor, CRFs will be collected and data will be entered onto a database using double independent data entry. The trial data will be stored in a computer database maintaining confidentiality in accordance with national data legislation.

In order to ensure data quality, a uniform hard copy CRF will be designed for use at all the sites. Data will then be sent to the Data centre for data entry, data cleaning and statistical analysis as defined in protocol and statistical analysis plan.

12.8. Confidentiality of trial documents and subjects records

The investigator must assure that subjects' anonymity will be maintained and that their identities are protected from unauthorized parties. On CRFs or other documents submitted to the sponsor, subjects should not be identified by their names, but exclusively by an identification code and initials. The investigator should keep a subject enrolment list showing codes, names, and addresses. The investigator should maintain documents for submission to sponsor authorized representative, and subject's signed written consent forms, in strict confidence.

13. Protocol Amendments

The Principal investigator will ensure that the study protocol is strictly adhered to throughout, and that all data are collected and recorded correctly on the CRF. The Principal investigator may contact the medical coordinator for a protocol waiver for minor deviations from the protocol, e.g. patient unable to attend during visit window.

All protocol modifications must be documented in writing. Any protocol amendment must be approved and signed by the sponsor and the Principal investigator and is to

be submitted to the appropriate IEC for information and approval in accordance with local requirements, and to regulatory agencies if required. Approval by IEC (and Regulatory Authority, if applicable) must be awaited before any changes can be implemented, except for changes necessary to eliminate an immediate hazard to trial subjects, or when the change involves only logistical or administrative aspects of the trial [e.g. change in clinical monitor[s], change of telephone number[s].

The protocol amendment can be initiated by either sponsor or by any Principal investigator.
The investigator will provide in writing the reasons for the proposed amendment and will discuss with the medical coordinator and sponsor.

14. Termination of the Study

Both the sponsor and the investigator reserve the right to terminate the study at any time prior to inclusion of the intended number of subjects, but they intend to exercise this right only for valid scientific or administrative reasons. Should this be necessary, both parties will arrange the procedures on an individual study basis after review and consultation. In terminating the study, the sponsor and the investigator will assure that adequate consideration is given to the protection of the subject’s interest.

Reasons for termination by the sponsor(s) may include but not be limited to:

- Too low enrolment rate.
- Protocol violations.
- Inaccurate or incomplete data.
- Unsafe or unethical practices.
- Questionable safety of the test article.
- Suspected lack of efficacy of the test article.
- Following the recommendation of the DSMB or IEC
- Administrative decision.

Reasons for termination by the investigator may be:

- Insufficient time or resource to conduct the study
- Lack of eligible patients

In the event that a study is terminated either by the sponsor or by the investigator, the investigator has to:

- Complete all CRFs to the greater extent possible
- Return all test articles, CRF, and related study materials to the sponsor who provided them
- Answer all questions of the sponsors or their representatives related to data of subjects enrolled at the site prior to study termination
- Ensure that subjects enrolled in the study who had not yet reached a follow up time point are followed up with the necessary medical care.

- Provide in writing the reasons for his decision to the national health authority and the sponsor.

15. Ethics

The experimental protocol for this study has been designed in accordance with the general ethical principles outlined in the Declaration of Helsinki and ICH guidelines for Good Clinical Practice (International Committee for Harmonization). DNDi assures that it will comply with all applicable state, local and foreign laws for protecting the rights and welfare of human subjects. This protocol and any protocol amendments will be reviewed / approved by an IEC before its implementation.

It is the responsibility of the Investigator to apply for review to the IEC of the country where the study takes place regarding local rules and regulations. Written approval from all involved IECs must be obtained before implementation of any protocol-specified intervention /investigation provided to the subject [such as subject information sheets or descriptions of the study].

Any modifications made to the protocol after receipt of the IEC approval must also be submitted by the investigator in writing to the IEC in accordance with local procedures and regulatory requirements.

15.1. Informed consent process

Inclusion in the study will occur only if the subject gives written informed consent. It is the responsibility of the investigator / designee to obtain written informed consent from each individual participating in this study, after adequate presentation of aims, methods, anticipated benefits, and potential hazards of the study. The written informed consent document will be translated into the local language or a language understood by the subject(s). If needed, the person will be given time to discuss the information received with members of the community or family before deciding to consent. The subject will be asked to provide written and signed consent.
If the subject is illiterate, a literate witness must sign (this person should have no connection to the research team, and, if possible, should be selected by the participant).

The informed consent is composed of 2 parts:

- Part A explains all the study related procedures (i.e. VL test treatment procedure and all follow-up assessments).
- Part B explains the secondary prophylaxis procedure.

Women of child bearing age who are not using an acceptable method of contraception (IUCD or Depo-Provera) will be requested to consider Depo-Provera as a method of contraception during and for 6 months after completion of the trial. They will be informed of the risks (and benefits) of its use and asked to consent to receiving Depo-Provera. The consent process will be similar to that for participation

in the clinical trial. If women of childbearing age do not consent to receive depo-provera and are not using another acceptable method of contraception they will be excluded from participation in the trial and treated with AmBisome 40 mg/Kg total dose.

It is not necessary to consent the patient for the pentamidine treatment at the time of inclusion into the trial because eligibility for the secondary prophylaxis will only be assessed after successful completion of VL treatment. This is done in order to bring as much clarity as possible to the patient.

If new safety information results in significant changes in the risk/benefit assessment, the consent form should be reviewed and updated if necessary. All subjects (including those already being treated) should be informed of the new information, given a copy of the revised form and give their consent to continue in the study.

15.2. Ethical aspects of subject inclusion and study procedures

The effective treatment of VL benefits not only the individual patient but also the community by reducing the reservoir of infection for onward transmission by the sandfly vector. The evaluation of new and better treatments for VL, particularly combination treatments, is anticipated to minimise the development of parasite resistance and will reduce hospitalization and public health costs.
Patients will experience some pain during splenic/bone marrow aspiration for parasitology and while blood is drawn during venipuncture. Local anaesthetic according to the routine practice for the management of patients will be used for bone marrow aspiration.

While the management of HIV-VL co-infected in general will be according to the Ethiopian Guidelines that are currently under revision for the treatment of Leishmaniasis, initiation of ART will be soon after start of treatment of VL, normally after 3 weeks, which is now current practice in both Gondar University hospital and the MSF treatment site at Abdurafi. In addition, based on current practice in other endemic areas secondary prophylaxis will be given to prevent relapse. Despite this, based on the literature a number of patients will inevitably experience relapse because of failure to mount an appropriate immune response. This mechanism is subject of a side study that is aimed to better understand these immune responses in order to develop a specific intervention. In addition, as the interaction between antiretroviral drugs and antileishmanial drugs as used in this study is largely unknown drug levels of miltefosine, Ambisome®, tenofovir, lamivudine and efavirenz will be measured.

The total volume of blood drawn, up to day 29 assessment, is 24mL and another total of 12mL (17mL for the extended treatment) will be drawn at different time points during the follow up period (from day 58 to 390). In case of extended treatment, another 5mL will be collected at day 58 assessment.
For the patients who will be studied for immunological markers and pharmacokinetics the maximum amount of blood taken up to day 29 assessment will be 63 mL. Another total of 30 mL will be collected at various time points until the last follow up

assessment (i.e. day 390). In case of extended treatment, another 6mL will be drawn for the pharmacokinetic study.

15.3. Ethical aspects of study treatments

Ambisome® has been widely used in many countries for more than 15 years for life-threatening infections including VL. It has also been used extensively in VL patients co-infected with HIV.It is generally considered to be well-tolerated and safe and is administered by intravenous infusion. It is associated with significantly less renal toxicity than the parent drug, amphotericin B. During infusion, patients frequently suffer chills, back pain and 'flu-like symptoms. These symptoms can be controlled with simple anti-inflammatory drugs such as paracetamol and can also be reduced by slowing the intravenous infusion rate.

Miltefosine was licensed for use in VL in 2002. It is known to be teratogenic in experimental animals and therefore women of child-bearing potential must be protected from pregnancy during and for 3 months after completion of treatment. It is an oral medication and the commonest side effects experienced during treatment are gastrointestinal; nausea, vomiting and diarrhoea. Only infrequently have these symptoms been sufficiently severe to require stopping treatment. In a phase-4 trial in India there were hepatic and renal toxicity observed mostly at CTC grade1 , but a few at CTC grade 3 (i.e AST: 1.2%; ALT: 0.7%; Creatinine 0.6%). The data on a phase-4 trial from Nepal indicate that the cure rate at 6 month follow up was 86% (95% CI = 78.4- 91.4), with 10.6 % failure and 3.6% LTFU. The major adverse events were those of GI reactions including vomiting, diarrhoea, and abdominal pain. All were of CTC grade 1 or 2. There was no loss of compliance due to side effects. There was one pregnancy (conception during treatment) where the baby was normal and was delivered at full term (Rijal, et al. Unpublished data). There were reversible gynecomastia in 2 patients.

Should women of the child-bearing age not be willing to use an approved method of contraception and undergo pregnancy test at the start of the study, they will be treated outside the trial according to the National VL treatment guidelines.

The antiretroviral drug regimen used in this study is a combination of tenofovir (TDF), lamivudine (3-TC) and efavirenz (EFV); in case a patient develops renal impairment, TDF will be replaced with zidovudine (AZT). These regimens are the currently recommended regimens according to the Ethiopian Guidelines for Antiretroviral Treatment.

15.4 Patient costs

Patients will be reimbursed for travel to and from the study site but will not receive any payment for trial participation. Any medication that is required during the trial period will be provided free of charge to the patient. Food during the in-patient treatment phase will also be provided free of charge to the patient. This is seen as an essential part of the patient care plan bearing in mind the high prevalence of malnutrition and the poverty of these patients.

16 Insurance and Liability

DNDi is insured to indemnify the investigator against claims arising from the trial,

except for claims that arise from malpractice and/or negligence.

17 Reporting and publication

All clinical trials will be registered with a recognised clinical trial registry such as www.clinicaltrials.gov.

Any reports including study results will be provided to the ethical and regulatory bodies according to local requirements.

References

[1] Cota, G.F., et al. Predictors of Visceral Leishmaniasis Relapse in HIV Infected Patients: A Systematic Review. *PLoS NTD*, 2011, 5(6): e1153.

[2] Mary C. et al., *Am J Trop Med Hyg.* 2006 Nov;75(5):858-63, Reference values for Leishmania infantum parasitemia in different clinical presentations: quantitative polymerase chain reaction for therapeutic monitoring and patient follow-up.

[3] WHO. Control of the Leishmaniasis: Report of the WHO expert committee on the Control of Leishmaniasis, Geneva, 22-26 March 2010. *WHO*, 2010.

[4] Ritmeijer K., et al. *Clin Infect Dis.* 2006 Aug 1;43(3):357-64. Epub 2006 Jun 20. A comparison of miltefosine and sodium stibogluconate for treatment of visceral leishmaniasis in an Ethiopian population with high prevalence of HIV infection.

[5] Ritmeijer K., et al., Limited effectiveness of high-dose liposomal amphotericin B (AmBisome) for treatment of visceral leishmaniasis in an Ethiopian population with high HIV prevalence. *Clin Infect Dis.* 2011 Dec;53(12):e152-8. Epub 2011 Oct 19.

[6] Seifert, K. and S.L. Croft, *Antimicrob Agents Chemother,* 2006. **50**(1): p. 73-9, In vitro and in vivo interactions between miltefosine and other antileishmanial drugs.

[7] Sundar S., et al. Comparison of short-course multidrug treatment with standard therapy for visceral leishmaniasis in India: an open-label, non-inferiority, randomised controlled trial. *Lancet*, 2011; 377: 477-86.

[8] Bellissant, E. *Stats in Med*, 1990, Vol 9, 907-917, Application of the triangular test to phase II cancer clinical trials.

[9] Ranque, S., et al., *Malaria Journal*, 2002, 1:13, Triangular test applied to the clinical trial of azithromycin against relapses in Plasmodium vivax infections.

[10] Bellissant E., et al., *American Journal of Clinical Oncology*, 1996, 19(4): 422-30, The group sequential triangular test for phase II cancer clinical trials.

[11] Alvar J. et al., *Clinical Microbiology Reviews* 21, 334–359, The relationship between leishmaniasis and AIDS: the second 10 years.

[12] Morales MA., et al., *J Infect Dis*. 2002 May 15;185(10):1533-7. Epub 2002 Apr 22., Relapses versus reinfections in patients coinfected with Leishmania infantum and human immunodeficiency virus type 1.

[13] Alvar, Clin Microbiol Review 2008.

[14] Nascimento ET, Moura ML, Queiroz JW, Barroso AW, Araujo AF, Rego EF, Wilson ME, Pearson RD, Jeronimo SM. The emergence of concurrent HIV-1/AIDS and visceral leishmaniasis in Northeast Brazil.Trans R Soc Trop Med Hyg. 2011 May; 105(5):298-300. Epub 2011 Apr 7.

[15] Hailu A, Gebre-Michael T, Berhe N & Balkew M (2006) Leishmaniasis in Ethiopia. *Epidemiology and Ecology of Healthand Disease in Ethipiopia. 1st edn* (eds Berhane Y, Hailemariam D & Kloos H)

Shama Press, Addis Ababa, pp. 615–634.

[16] Ritmeijer K. et al., *Trans R Soc Trop Med Hyg.* 2001 Nov-Dec;95(6):668-72. Ethiopian visceral leishmaniasis: generic and proprietary sodium stibogluconate are equivalent; HIV co-infected patients have a poor outcome.

[17] Lyons S, Veeken H, Long J., *Trop Med Int Health*. 2003 Aug;8(8):733-9., Visceral leishmaniasis and HIV in Tigray, Ethiopia.

[18] Nascimento ET, Moura ML, Queiroz JW, Barroso AW, Araujo AF, Rego EF, Wilson ME, Pearson RD, Jeronimo SM., *Trans R Soc Trop Med Hyg*. 2011 May;105(5):298-300. Epub 2011 Apr 7., The emergence of concurrent HIV-1/AIDS and visceral leishmaniasis in Northeast Brazil.

[19] Hurissa Z. et al., *Trop Med Int Health*. 2010 Jul;15(7):848-55. Epub 2010 May 14., Clinical characteristics and treatment outcome of patients with visceral leishmaniasis and HIV co-infection in northwest Ethiopia.

[20] Barat C, Zhao C, Ouellette M, Tremblay MJ., *J Infect Dis*. 2007 Jan 15;195(2):236-45. Epub 2006 Dec 13., HIV-1 replication is stimulated by sodium stibogluconate, the therapeutic mainstay in the treatment of leishmaniasis.

[21] Hailu A. et al., *PLoS Negl Trop Dis.* 2010 Oct 26;4(10):e709. Geographical variation in the response of visceral leishmaniasis to paromomycin in East Africa: a multicentre, open-label, randomized trial.

[22] Laguna F. et al. ,*AIDS*. 1999 Jun 18;13(9):1063-9. Treatment of visceral leishmaniasis in HIV-infected patients: a randomized trial comparing meglumine antimoniate with amphotericin B. Spanish HIV-Leishmania Study Group.

[23] Delgado J. et al., *Am J Trop Med Hyg.* 1999 Nov;61(5):766-9. High frequency of serious side effects from meglumine antimoniate given without an upper limit dose for the treatment of visceral leishmaniasis in human immunodeficiency virus type-1-infected patients.

[24] Laguna F. et al., *J Antimicrob Chemother*. 2003 Sep;52(3):464-8. Epub 2003 Jul 29. Amphotericin B lipid complex versus meglumine antimoniate in the treatment of visceral leishmaniasis in patients infected with HIV: a randomized pilot study.

[25] Pintado V, Martín-Rabadán P, Rivera ML, Moreno S, Bouza E. *Medicine* (Baltimore). 2001 Jan;80(1):54-73 Visceral leishmaniasis in human immunodeficiency virus (HIV)-infected and non-HIV-infected patients. A comparative study.

[26] Lopez-Velez R. et al., *Am J Trop Med Hyg.* 1998 Apr;58(4):436-43. Clinicoepidemiologic characteristics, prognostic factors, and survival analysis of patients coinfected with human immunodeficiency virus and Leishmania in an area of Madrid, Spain.

[27] Davidson RN, Russo R. *Clin Infect Dis.* 1994 Sep;19(3):560. Relapse of visceral leishmaniasis in patients who were coinfected with human immunodeficiency virus and who received treatment with liposomal amphotericin B.

[28] Russo R. et al., *J Infect.* 1996 Mar;32(2):133-7. Visceral leishmaniasis in HIV infected patients: treatment with high dose liposomal amphotericin B (AmBisome).

[29] Berhe N, et al., HIV viral load and response to antileishmanial chemotherapy in co-infected patients. *AIDS*. 1999 Oct 1;13(14):1921-5.

[30] Sindermann H. Et al., *Clin Infect Dis.* 2004 Nov 15;39(10):1520-3. Epub 2004 Oct 18. Oral miltefosine for leishmaniasis in immunocompromised patients: compassionate use in 39 patients with HIV infection.

[31] Sinha PK, et al., *Clin Infect Dis.* 2011 Oct;53(7):e91-8, Liposomal amphotericin B for visceral leishmaniasis in human immunodeficiency virus-coinfected patients: 2-year treatment outcomes in Bihar, India,

[32] Ritmeijer K., data presented at ECTMIH, Barcelona, Oct 2011

[33] Mueller Y. et al., *Trop Med Parasitol*. 2008 Jan;102(1):11-9., Safety and effectiveness of amphotericin B deoxycholate for the treatment of visceral leishmaniasis in Uganda.

[34] Ritmeijer K., pers comm.

[35] Bekersky I. et al., *Pharm. Res*. 1999; 16:1694-1701, Safety and toxicokinetics of intravenous liposomal amphotericin B (AmBisome) in beagle dogs.

[36] Hughes CA, Foisy M, Tseng A. Interactions between antifungal and antiretroviral agents. *Expert Opin Drug Saf* 2010; 9:723-742.

[37] Inselmann G, Volkmann A, Heidemann HT. Comparison of the effects of liposomal amphotericin B and conventional amphotericin B on propafenone metabolism and hepatic cytochrome P-450 in rats. Antimicrob. *Agents Chemother* 2000; 44:131-133.

[38] Brockmeyer NH. et al., *Eur. J. Med. Res* 2004; 9:51-54, Impact of amphotericin B on the cytochrome P450 system in HIV-infected patients.

[39] Bellmann R. *Curr Clin Pharmacol* 2007; 2:37-58, Clinical pharmacokinetics of systemically administered antimycotics.

[40] German Drug Registration Authorities. Impavido 10/50 mg Kapseln- Fachinformation. 2008; Available at: http://www.pharmnet-bund.de/dynamic/de/index.html. Accessed 15 February 2010.

[41] Dorlo TPC, Tellingen O, De Vries PJ, Beijnen JH. The Role of ABC Transporters in the Pharmacokinetics of Miltefosine. *4th Worldleish Congress on Leishmaniasis 2009*, Lucknow, India: Available at: http://www.worldleish4.org/session_details.php?sessionId=678. Accessed 13 October 2011.

[42] Weiss J. et al., *European Journal of Pharmacology* 2008; 579:104-109, Comparison of the induction of P-glycoprotein activity by nucleotide, nucleoside, and non-nucleoside reverse transcriptase inhibitors.

[43] Article just submitted to *Journal of Antimicrobial Chemotherapy*

Thomas P.C. Dorlo et al., Translational pharmacokinetic modelling and simulation for the assessment of duration of contraceptive cover after use of miltefosine for the treatment of visceral leishmaniasis
