# Supplementary material for: A randomized trial of AmBisome monotherapy and AmBisome and miltefosine combination to treat visceral leishmaniasis in HIV co-infected patients in Ethiopia
Source: PLoS Negl Trop Dis. 2019 Jan 17;13(1):e0006988. doi: 10.1371/journal.pntd.0006988 (PMC6336227; doi:10.1371/journal.pntd.0006988)
Supplement: S1 Approval — (PDF) [file pntd.0006988.s002.pdf]

ጎንደር ዩኒቨርስቲ
ምርምርና ህብረተሰብ አገልግሎት ምክትል
ፕሬዚዳንት ጽ/ቤት፤ ጎንደር፤ ኢትዮጵያ

University of Gondar
Office of Vice President for Research &
Community Service, Gondar, Ethiopia

Rfe. R/C/S/V/P/05/ 376 /2013

Date: 18/04/ ,2013 .

To: Dr. Ermias Diro
Department of Internal Medicine
University of Gondar

Subject: - Ethical Clearance

Your research project proposal titled **"A randomized trial of AmBisome monotherapy and combination of AmBisome and miltefosine for the treatment of VL in HIV positive patients in Ethiopia followed by secondary VL prophylactic treatment with pentamidine."** has been reviewed by the Institutional Ethical Review Board of University of Gondar for its Ethical soundness, and it is found to be ethically acceptable.

Thus, the Research and Community Service Vice President Office has awarded this ethical clearance for the above stated study to be carried out by Dr. Ermias Diro as principal investigator and Professor Asrat Hailu as Co-investigator as of April 12, 2013.

These investigators are expected to submit their research progress report to the Research and Community Service Vice President Office of the University of Gondar.

Best regards,

Dr. Afework Kassu
V/President for Research & Community Service

Office of the President
University of Gondar
