# Supplementary material for: A randomized trial of AmBisome monotherapy and AmBisome and miltefosine combination to treat visceral leishmaniasis in HIV co-infected patients in Ethiopia
Source: PLoS Negl Trop Dis. 2019 Jan 17;13(1):e0006988. doi: 10.1371/journal.pntd.0006988 (PMC6336227; doi:10.1371/journal.pntd.0006988)
Supplement: S2 Approval — (PDF) [file pntd.0006988.s003.pdf]

በኢትዮጵያ ፌዴራላዊ ዴሞክራሲያዊ ሪፐብሊክ
የሳይንስና ቴክኖሎጂ ሚኒስቴር
The Federal Democratic Republic of Ethiopia
Ministry of Science and Technology

ቁጥር 3.10/454/05
Ref. No.
ቀን 15-06-2005
Date

**To: University of Gondar College of Medicine and Health science**

**Addis Ababa**

**Re: A Randomized Trial Of Ambisome ®Monotherapy And Combination Of Ambisome® and Miltefosine for the treatment of VL in HIV positive patients in Ethiopia Followed By Secondary VL Prophylcatic Treatment With Pentamidine**

Dear sir/Mr./s/Dr.

The National Research Ethics Review committee (NRERC) has reviewed the aforementioned project protocol in an expedited manner. We are writing to advise you that NRERC has granted

*Full Approval*

To the above named project, for a period of **one year (February 22, 2013- February 21, 2014).** All your most recently submitted documents have been approved for use in this study.

The study should comply with the standard international and national scientific and ethical guidelines. Any change to the approved protocol or consent material must be reviewed and approved through the amendment process prior to its implementation. In addition, any adverse or unanticipated events should be reported within 24-48 hours to the NRERC. Please ensure that you submit progress report once in a four month and annual renewal application 30 days prior to the expiry date.

We, therefore, request your esteemed organization to ensure the commencement and conduct of the study accordingly and wish for the successful completion of the project.

With regards,

*Yohannes Sitotaw*

*Secretary of NRERC*

Cc _ Dr Ermias Diro (PI)

ማነጋገር ቢያስፈልግዎ
You may Contact

ፖ.ሳ.ቁ. P.O.Box 2490
አዲስ አበባ ኢትዮጵያ Addis Ababa, Ethiopia
E-mail most@ethionet.et
ስልክ Tel. 251-011-4-674353
Web site:-http://www.most.gov.et
ፋክስ Fax +251-011-4-66 02 41
