# Supplementary material for: A randomized trial of AmBisome monotherapy and AmBisome and miltefosine combination to treat visceral leishmaniasis in HIV co-infected patients in Ethiopia
Source: PLoS Negl Trop Dis. 2019 Jan 17;13(1):e0006988. doi: 10.1371/journal.pntd.0006988 (PMC6336227; doi:10.1371/journal.pntd.0006988)
Supplement: S1 Table — (DOCX) [file pntd.0006988.s005.docx]

S5 Rescue Medication

Table 1: Rescue medication received during the 1-year follow up of the HIV-VL0511 trial

| **Treatment received** | **Number of rescue treatment course** |
| --- | --- |
| AmBisome® / Miltefosine | 44 |
| Sodium Stibogluconate | 6 |
| AmBisome® | 3 |
| AmBisome® / Miltefosine / Itraconazole | 3 |
| Sodium Stibogluconate / Paromomycin | 3 |
| Sodium Stibogluconate / AmBisome® | 1 |
| AmBisome® / Miltefosine / Pentamidine | 1 |
